# Systemic therapy in the treatment of recurrent or refractory intracranial meningiomas: A systematic review and individual patient data meta-analysis

**DOI:** 10.1007/s10143-026-04233-w

**Published:** 2026-04-16

**Authors:** Emmanuel O. Mensah, Arnab Ghosh, Aditi Rane, Justin Kim, Pratham B. Bhatt, Andrew F. Alalade

**Affiliations:** 1 Department of Neurosurgery, Massachusetts General Hospital, Boston, MA USA; 2 School of Clinical Medicine, University of Oxford, Oxford, UK; 3 School of Medicine, University of Lancashire, Preston, PR1 7BH UK; 4 Harvard Medical School, Boston, MA USA; 5 Chobanian & Avedisian School of Medicine, Boston University, Boston, MA USA; 6 Department of Neurosurgery, Royal Preston Hospital, Lancashire Teaching Hospitals NHS Foundation Trust, Preston, PR2 9HT UK

**Keywords:** Meningioma, Treatment-refractory, Progressive, Chemotherapy, Kaplan-Meier survival

## Abstract

Recurrent or refractory meningiomas pose major therapeutic challenges once surgical and radiotherapeutic options are exhausted. The role and comparative effectiveness of systemic pharmacologic therapies remain unclear. This study evaluated survival outcomes and toxicity profiles of systemic agents used in adults with recurrent intracranial meningiomas. A systematic search of PubMed, Embase, and Web of Science (through November 2024) followed PRISMA-IPD guidelines. Eligible studies included adults with WHO grades I-III meningiomas treated with systemic agents for recurrent disease and reporting reconstructable time-to-event data. Individual patient data and digitized Kaplan–Meier estimates were pooled. Outcomes included progression-free survival (PFS), overall survival (OS), and treatment-related toxicity. Twenty-five studies (484 patients) were included. Across systemic agents, outcomes predominantly reflected disease stabilization rather than objective tumor regression. Hydroxyurea (*n* = 8) was most frequently evaluated, followed by bevacizumab (5), somatostatin analogues (5), interferon-α (3), PD-1/PD-L1 inhibitors (2), and tyrosine kinase inhibitors (2). Median PFS varied widely by WHO grade, with longer PFS generally observed in grade 1 tumors. Interferon-α and somatostatin analogs demonstrated longer PFS in grade 1 compared with higher-grade tumors Median OS ranged from 8 to 32 months without grade-specific differences. Toxicity profiles differed by agent: hydroxyurea was associated with frequent hematologic adverse effects (67.4%,), while bevacizumab and somatostatin analogs were generally well tolerated with low discontinuation rates. Hydroxyurea, interferon-α, and somatostatin analogues offer modest disease stabilization, particularly in lower-grade tumors, with generally manageable toxicity. These findings provide pooled, non-comparative benchmark reference ranges for future systemic therapy trials in recurrent/refractory meningioma.

**Supplementary Information:**

The online version contains supplementary material available at 10.1007/s10143-026-04233-w.

## Introduction

Meningiomas are the most common primary intracranial tumors in adults, comprising approximately 57.4% of all non-malignant and 42.6% of primary central nervous system neoplasms [1]. Although most are histologically benign (WHO grade 1) and curable with surgery, a subset displays atypical or anaplastic features (WHO grades 2–3) associated with higher recurrence and poorer survival [2]. Standard management for grades 2–3 meningiomas consists of maximal safe resection followed by adjuvant radiotherapy, with recurrence rates of approximately 30–60% in grade 2 tumors within 10 years and over 60–80%in grade 3 tumors within 5 years despite aggressive local therapy [3]. Correspondingly, 5-year overall survival (OS) falls from approximately 75–80% in grade 2 to 40–50% in grade 3 disease, with median progression-free survival (PFS) frequently under 3 years [4].

While local salvage with repeat resection or re-irradiation remains standard in patients with recurrent disease, feasibility declines with successive treatments due to anatomical and cumulative dose constraints. Effective systemic therapies are limited and largely considered investigational or palliative [5, 6].

Prior meta-analyses of systemic therapy in meningioma are limited by small, heterogeneous cohorts, precluding grade- or drug-specific survival estimates, while inconsistent toxicity reporting has hindered balanced evaluation of benefit versus treatment burden [7, 8]. Consequently, clinicians lack reliable pooled benchmarks for PFS and OS to guide counselling and trial design. This systematic review and individual patient data (IPD) meta-analysis pools reconstructed and reported patient-level data to harmonize estimates of PFS, OS, and treatment-related toxicity across WHO grades and drug classes, thereby establishing non-comparative benchmarks.

## Methods

This systematic review was conducted in accordance with the Preferred Reporting Items for Systematic Reviews and Meta-Analyses of individual participant data (PRISMA-IPD) guidelines and prospectively registered with PROSPERO (ID: CRD42025602986) [9].

### Literature search

A comprehensive search of the MEDLINE/PubMed, Embase, and Web of Science databases was performed from inception to November 19, 2024, using combinations of: (“meningioma”) AND (“recurrent” OR “refractory” OR “resistant” OR “unresectable” OR “progressive)” AND (“chemotherapy” OR “systemic therapy” OR “targeted therapy” OR “immunotherapy” OR “drug”). Two independent reviewers screened titles and abstracts, followed by full-text review of potentially eligible studies. Conflicts were resolved through discussion with a senior reviewer. Reference lists of included articles were manually screened to ensure completeness.

### Study selection criteria

Inclusion criteria included studies of adults with intracranial meningioma reported as WHO grade 1–3 intracranial meningioma (or equivalent historical terminology: benign, atypical, or aplastic) treated for recurrent or refractory disease; use of systemic therapies, including combination regimens where the systemic agent was the primary exposure; adequate time-to-event PFS and OS outcomes with sufficient detail to reconstruct of Kaplan-Meier estimates from data (time-to-event and censoring) or published curves/tables with risk data; at least two studies reporting extractable data; and a combined sample size of ≥ 5 patients per study. Recurrent, refractory, or progressive meningioma was defined pragmatically as post-local therapy (surgery and/or radiotherapy) tumor progression or recurrence prompting systemic therapy.

Exclusion criteria included studies < 5 patients; studies without extractable individual patient-level data; conference abstracts; animal or pediatric studies; and non-pharmacologic interventions. No restrictions were placed on language or geographic setting, with non-English full texts translated for eligibility assessment when encountered.

### Data extraction

Three reviewers independently extracted data from eligible studies using a standardized form, with disagreements resolved by consensus or adjudication by a senior reviewer. Extracted information included study characteristics, patient demographics and clinical features (age, sex, performance status as measured by Karnofsky Performance Scale [KPS] or Eastern Cooperative Oncology Group [ECOG], and meningioma grade and location), and treatment details (prior local therapies, systemic agent, and treatment regimen). Outcomes of interest were PFS, OS, and toxicity data (classified as hematologic and non-hematologic events). When survival outcomes were presented graphically, time-to-event data were digitized using WebPlotDigitizer (v4.0) (Supplementary Methods).

### Quality assessment

Risk of bias was evaluated using the Cochrane ROBINS-I tool by two independent reviewers, with disagreements resolved through consensus [10].

### Statistical analysis

PFS was the primary endpoint, defined as the time from initiation of systemic therapy to tumor progression or death. OS was analyzed when reported. Survival distributions were estimated using the Kaplan-Meier method with log–log 95% confidence intervals (CIs), with median survival times calculated overall and stratified by WHO grade; “NA” denoted medians not reached due to insufficient events. Grade-based comparisons used log-rank tests for global and pairwise contrasts. Effect sizes were estimated using Cox proportional hazards models, reported as hazard ratios (HRs) with 95% CIs.

Landmark PFS estimates at 6, 12, 18, and 24 months were estimated by grade when at least two studies with a combined sample of ≥ 10 patients provided reconstructable time-to-event data for a given agent. Pairwise comparisons at each landmark used Wald-type tests on the complementary log–log scale with Holm correction for multiple testing and were interpreted cautiously given heterogeneity in progression criteria and imaging schedules. Additionally, grade 2/3 tumors were collapsed and compared with grade 1 using Kaplan-Meier curves, log-rank tests, and Cox regression.

Toxicity outcomes were analyzed as hematologic and non-hematologic events, stratified by grade (1/2 vs. ≥3) and reported per patient or per treatment cycle. Given variability in toxicity grading systems (CTCAE versions 1.0–5.0 and WHO criteria), data were harmonized by mapping WHO grades to corresponding CTCAE grades when cutoffs matched. For each drug, proportions of patients or cycles with any toxicity, grade 1/2 events, grade ≥ 3 events, and treatment discontinuation due to toxicity were extracted, and single-arm meta-analyses were performed to generate pooled estimates with 95% CIs.

All analyses were performed in R (version 4.4.2, The R Foundation for Statistical Computing) using the *survival* and *survminer* packages. Statistical significance was defined as a two-tailed *p* < 0.05.

## Results

### Study selection

**Fig. 1:**
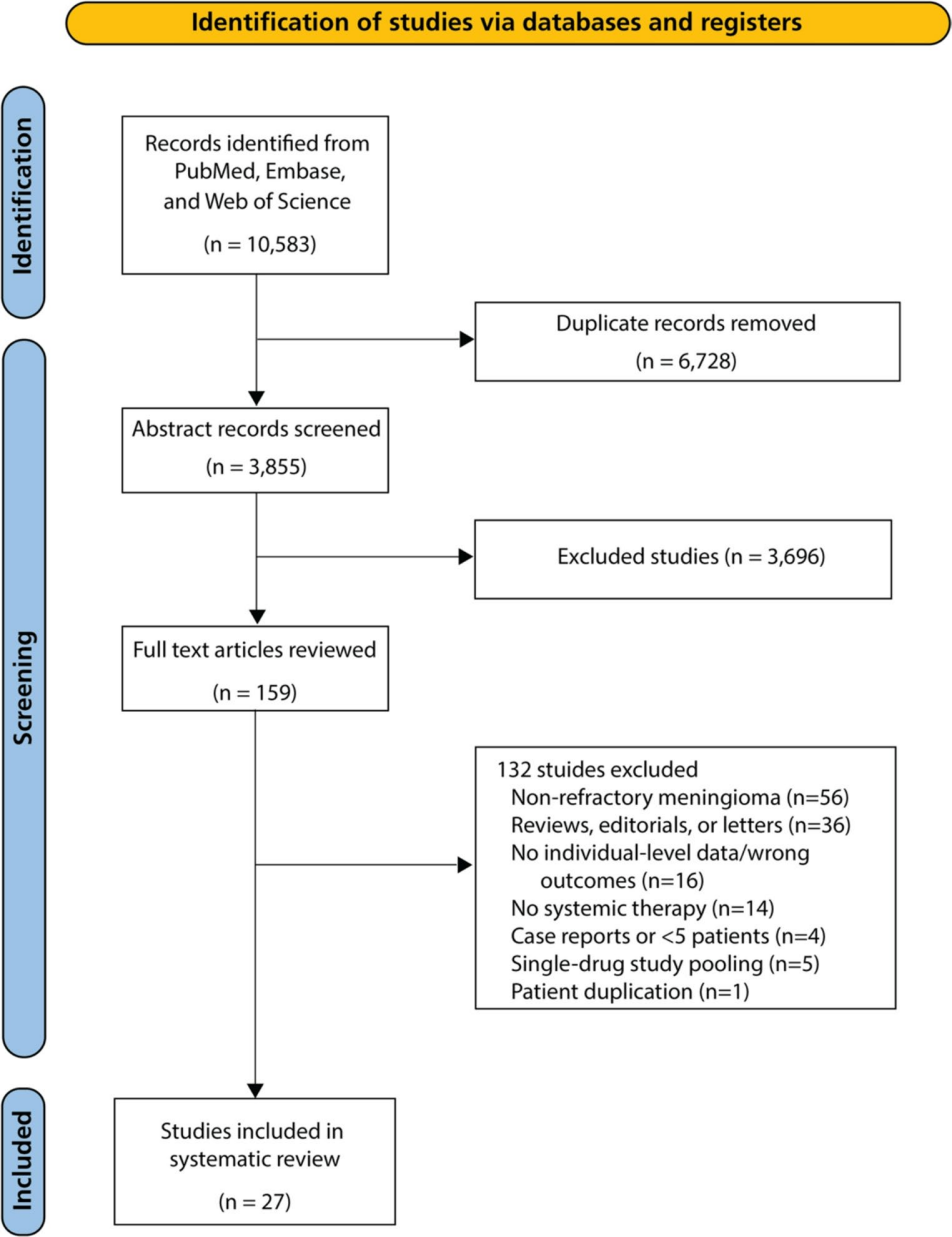
PRISMA

Database search identified 10,583 records. After deduplication, 3,855 unique titles and abstracts were screened. Of 228 full-text articles assessed, 25 studies met inclusion criteria and were included in the final analysis (Fig. 1). Notably, one hydroxyurea study was excluded for patient duplication [11].

### Study characteristics

Twenty-seven studies comprising 511 patients treated for recurrent or refractory meningioma were included (Tables 1 and 2) [12–38]. Studies were published between 1997 and 2022 across 7 countries, predominantly from the United States (19, 70.4%). Sample sizes ranged from 6 to 35 patients (median, 16). The cohort included 190 males (37.2%), and 296 females (57.9%), nd 25 unreported (5.2%), with a median age of 60 years (range: 20 to 89). Nine studies (33%) were retrospective, while 18 studies (67%) were prospective (including phase II trials). Performance status (KPS or ECOG) was reported in 10 studies [13, 14, 17, 24, 27, 28, 31, 34–36]. WHO grade distribution was variable reported: 20 studies included grade 1, 19 included grade 2, and 13 included grade 3 meningiomas, with some combining grades 2 and 3 [16, 21, 29]. Tumors were located in the convexity (49.7%), skull base (38.6%), or both locations (9.6%).

**Table 1:** Characteristics of included studies

Study	Country	Type of Study	Patients (Sex)	Age (median, range)	Performance status, median (range)	Grade distribution	Tumor location	Immuno-chemistry	Data source	Radiological criteria
Bevacizumab										
Lou et al. [24]	USA	Retrospective	14 (6 M, 8 F)	53.5 (20–70)	KPS, 80 (60–90)	Grade 1: 5 Grade 2: 5 Grade 3: 3	–	–	Mixed	RANO; MRI every 8 weeks
Nayak et al. [25]	USA	Retrospective	15 (7 F, 8 M)	55 (34–81)	–	Grade 2: 6 Grade 3: 9	–	–	Mixed	RANO; interval NR
Shih et al. [31]	USA	Phase II multicenter	16 (8 M, 8 F)	59 (29–84)	ECOG, 1 (0–3)	Grade 1: 4 Grade 2: 7 Grade 3: 5	–	–	Mixed	MacDonald criteria; MRI every 8 weeks
Alexander et al. [33]	USA	Retrospective	23 (10 F, 13 M)	55 (13–72)	–	Grade 1: 2 Grade 2: 9 Grade 3: 10	–	–	Mixed	NA; Imaging interval NR
Kumthekar et al. [36]	USA	Phase II multicenter	42 (22 M, 20 F)	57 (26–81)	KPS, 80 (60–100)	Grade 1: 10 Grade 2: 18 Grade 3: 10	–	VEGF VEGFR2 HER2	Mixed	MacDonald criteria; MRI every 8–12 weeks
Hydoxyurea										
Mason et al. [13]	Canada	Prospective multicenter	20 (9 M, 11 F)	59 (31–75)	KPS, 80 (50–100)	Grade 1: 16 Grade 2: 3 Grade 3: 1	Convexity: 2 Skull base: 6 Multiple: 12	–	Mixed	MRI every 3–6 months
Loven et al. [14]	Israel	Prospective	12 (5 M, 7 F)	52.5 (41–77)	ECOG, 1.5 (1–3)	Grade 1: 8 Grade 2: 4	Convexity: 7 Skull base: 5	–	IPD only	CT and Tl-201 SPECT; every 3 months
Newton et al. [15]	USA	Prospective	20 (4 M, 16 F)	59 (33–74)	–	Grade 1: 15 Grade 2: 1 NR: 4	Convexity: 6 Skull base: 14	–	IPD only	MacDonald criteria; MRI every 16 weeks
Hahn et al. [16]	Germany	Prospective	21 (7 M, 14 F)	60 (34–76)	–	Grade 1: 13 Grade 2/3: 4	Convexity: 2 Skull base: 14 Multiple: 1	–	IPD only	MacDonald-type; CT/MRT scans q3-6 months then q6-12 months
Chamberlain. [21]	USA	Retrospective	35 (10 M, 25 F)	63 (34–86)	–	Grade 2: 22 Grade 3: 13	Convexity: 20 Skull base: 8 Multiple: 7	–	Mixed	Macdonald criteria; MRI every 8 weeks
Kim et al. [23]	Korea	Retrospective	13 (4 M, 9 F)	61.7 (32–83)	–	Grade 1: 8 Grade 2: 5	Convexity: 7 Skull base: 6	–	IPD only	Macdonald criteria; MRI every 3–6 months (then yearly)
Karsy et al. [30]	USA	Prospective open-label Phase I/II	7 (1 M, 6 F)	56 (26–76)	–	Grade 1: 2 Grade 2: 5	–	–	Mixed	Macdonald criteria; MRI every 3 months
Kim et al. [32]	Korea	Retrospective	24 (11 M, 13 F)	55.7 (26.4–73.8)	–	Grade 2: 24	Convexity: 19 Skull base: 5	Ki-67 Mitotic index p53	Mixed	Macdonald criteria; MRI every 12 weeks
INF-α										
Kaba et al. [12]	USA	Prospective	6 (4 M, 2 F)	55 (48–80)	–	Grade 1: 2 Grade 2: 1 Grade 3: 3	–	–	IPD only	MRI every 8 weeks
Chamberlain and Glantz, [18]	USA	Prospective Phase II	35 (6 M, 29 F)	61 (36–88)	–	Grade 1: 35	Convexity: 20 Skull base: 10 Multiple: 5	–	Mixed	Macdonald criteria; MRI every 12 weeks
Chamberlain. [26]	USA	Retrospective	35 (17 M, 28 F)	63 (36–86)	–	Grade 2: 22 Grade 3: 13	Convexity: 25 Skull base: 9 Multiple: 1	–	Mixed	Macdonald criteria; MRI every 8 weeks
Somatostatin analogs										
Chamberlain et al. [17]	USA	Prospective multicenter	16 (5 M, 11 F)	61.5 (26–87)	KPS, 80 (50–90)	Grade 1: 8 Grade 2: 3 Grade 3: 5	Convexity: 6 Skull base: 6 Multiple: 4	SSTR positivity confirmed (111In-octreotide SPECT)	IPD only	MacDonald-type; MRI every 3 months
Johnson et al. [19]	USA	Prospective Phase II	11 (8 M, 3 F)	52 (35–65)	–	Grade 1: 3 Grade 2: 3 Grade 3: 5	–	SSTR2A (subset of patients)	Mixed	MacDonald-type; MRI/CT every 3 months
Schulz et al. [20]	Germany	Prospective	8 (1 M, 7 F)	52 (37–63)	–	Grade 1: 8	Skull base: 8	SSTR-positive tumors confirmed by octreotide scintigraphy	IPD only	Not specified; MRI every 12 months
Simo et al. [28]	Spain	Prospective Phase II	9 (8 M, 1 F)	65 (23–77)	KPS, 80 (60–100)	Grade 2: 5 Grade 3: 4	–	SSTR-positive tumors confirmed by octreotide SPECT	Mixed	MacDonald-type; contrast-enhanced MRI every 3 months
Norden et al. [29]	USA	Prospective phase II	34 (17 M, 17 F)	54 (36–81)	–	Grade 1: 16 Grade 2/3: 18	–	SSTR1/2A/3/5	KM only	MacDonald; MRI q2 months then q3 months
Temozolomide										
Chamberlain (2003)	USA	Prospective phase II	16 (5 M, 11 F)	62.5 (48–70)	–	Grade 1: 16	Convexity: 12 Skull base: 4	–	Mixed	Standard neuroradiographic response criteria (MacDonald-type; not explicitly named); Contrast-enhanced MRI q10 weeks
Belanger (2022)	USA	Retrospective	11 (4 M, 7 F)	56 (22–82)	–	Grade 1: 2 Grade 2: 7 Grade 3: 2	–	–	KM only	Not specified; MRI interval NR
PD-1/PD-L1 blockers										
Bi et al. [34]	USA	Prospective phase II open label	25 (9 M, 16 F)	60 (25–88)	KPS, 80 (70–100)	Grade 2: 18 Grade 3: 7	Convexity: 15 Skull base: 8 Multiple: 2	PD-1/PD-L1 IHC and TMB analysis	KM only	RANO; MRI every 8 weeks
Brastianos et al. [35]	USA	Prospective phase II open label	26 (15 M, 11 F)	61.4 (19–89)	ECOG, 0 (0–1)	Grade 2: 22 Grade 3: 3	–	PD-L1	KM only	RANO; MRI every 6 weeks
Tyrosine kinase inhibitors										
Horak et al. [22]	Austria	Retrospective	9 (4 M,5 F)	54 (42–74)	–	Grade 1: 1 Grade 2: 2 Grade 3: 6	–	PDGFRα/β c-Kit c-Abl Arg	IPD only	RECIST; MRI every 3 months
Raizer et al. [27]	USA	Prospective phase II	25 (15 M, 10 F)	59 (30–89)	KPS, 80 (60–100)	Grade 1: 2 Grade 2: 14 Grade 3: 8	–	–	Mixed	MacDonald; MRI every 8 weeks

Immunochemistry refers to tumor biomarker testing or receptor assessment performed in included patients (e.g., somatostatin receptor imaging or immunohistochemistry, PD-1/PD-L1 expression, VEGF pathway markers, Ki-67, or PDGFR expression). Data sources were classified as individual patient data (IPD), Kaplan–Meier survival data (KM), or mixed sources. NR indicates not reported. RANO = Response Assessment in Neuro-Oncology; RECIST = Response Evaluation Criteria in Solid Tumors; KPS = Karnofsky Performance Status; ECOG = Eastern Cooperative Oncology Group performance status; IHC = immunohistochemistry; SSTR = somatostatin receptor; PD-L1 = programmed death-ligand 1; PD-1 = programmed death receptor 1; TMB = tumor mutational burden

**Table 2:** Treatment characteristics

Study	Previous treatment									Current Treatment regimen	
	Previous surgery			Radiation			SRS	Systemic therapy			
	Patients	Count	Type	Patients	Count	Dose	Patients	Patients	Drugs		
Bevacizumab											
Lou et al. [24]	13/13	2	–	9/13	–	–	6/13	10/13	Etoposide (1), hydroxyurea (6), imatinib (6), octreotide (3), pasireotide (1), tamxofien (1), temozolomide (7)	–	
Nayak et al. [25]	15/15	3	GTR (5), STR (5), NA (5)	10/10	–	54 Gy	5/15	7/15	Hydroxyurea (3), imatinib (1), octreotide (2), pasireotide (1), sunitinib (2)	10 mg/kg q2w IV. Med cycle 9 (range 1–19)	
Shih et al. [31]	16/16	2	GTR (3), STR (13)	12/16	–	–	9/16	3/16	Hydroxyurea (3), octreotide (1)	10 mg/kg q2w IV and PO everolimus 10 mg OD	
Alexander et al. [33]	21/21	3	GTR (6), STR (12)	21/21	3	–	0/21	0/21	–	7.5, 10, or 15 mg/kg every 2–3 weeks.	
Kumthekar et al. [36]	33/42	3	–	25/42	1	–	22/42	13/42	Hydroxyurea (7), INF-a (1), octreotide (4), PTK787 (2), sunitinib (1), tamoxifen (1), temozolomide (1)	10 mg/kg first dose, q2w for 6 months, then q3w at 15 mg/kg.	
Hydroxyurea											
Mason et al. [13]	20/20	2	–	6/20	–	–	3/20	–	–	20–30 mg/kg/day, usually 1000 to 1500 mg OD	
Loven et al. [14]	12/12	–	–	5/12	–	–	1/1	–	–	20 mg/kg/day	
Newton et al. [15]	17/20	1	–	11/20		–	–	–	–	20 mg/kg/day	
Hahn et al. [16]	17/21	2	GTR (4), STR (13)	21/21	–	55.8 Gy	–	2/21	Hydroxyurea (2)	1500 mg/day, reduced to 1000 mg/day in patients ≥ 65 and those previously treated with CT	
Hydroxyurea											
Chamberlain. [21]	35/35	2	GTR (17), STR (13), Biopsy (5)	34/35	–	60 Gy	35/35	–	–	1000 mg/m^2^ PO BD every 4–6 weeks	
Kim et al. [23]	13/13	1	–	1/13	–	–	–	–	–	1000 mg/m^2^/day BD PO	
Karsy et al. [30]	7/7	–	GTR (4), STR (3)	5/7	–	–	5/7	2/7	Celecoxib (1), octreotide (1)	Hydroxyurea: 20 mg/kg/day, typically 1000 or 1500 mg/day. Verapamil SR: 120 mg/day 2/52, 240 mg/day 2/52, 360 mg/day 2/52, and 240 mg BD	
Kim et al. [32]	24/24	1	STR (24)	0/24	–	–	0/24	–	–	1000 mg/m^2^/day every 4 weeks	
INF-α											
Kaba et al. [12]	6/6	3	STR (2)	3/6	–	–	1/5	3/6	Adriamycin (1), dacarbazine (1), ifosfamide (1), mifepristone (2)	4 mU/m^2^/day, 5/7 in five patients; 5 mU/m^2^/day, 3/7 in one patient	
Chamberlain and Glantz, [18]	35/35	2	GTR (9), STR (21), Biopsy (5)	9/35	–	–	22/35	34/35	Bevacizumab (1), celecoxib (1), capecitabine (6), hydroxyurea (19), somatostatin (2), temozolomide (8)	10 mU/m^2^ SC every other day, reduced in 25% increments with toxicity	
Chamberlain. [26]	35/35	2	GTR (15), STR (16), Biopsy (4)	35/35	–	–	35/35	35/35	Hydroxyurea (35), somatostatin (1)	10 mU/m^2^ SC every other day, reduced in 25% increments with toxicity	
Somatostatin analogs											
Chamberlain et al. [17]	14/16	2	GTR (5), STR (7), Biopsy (2)	9/16	–	–	7/16	12/16	Celecoxib (1), erlotinib (1), hydroxyurea (8), IFN-a (1), tamoxifen (1), temozolomide (1), thalidomide (1)	30 mg IM every 28 days	
Johnson et al. [19]	11/11	–	–	9/11	–	–	–	3/11	Carmustine (1), hydroxyurea (1), tamoxifen (1)	150 mcg BD on day 1, 250 mcg BD on day 2, 500 mcg TDS thereafter	
Schulz et al. [20]	8/8	2	–	0/8	–	–	0/8	0/8	–	Sandostatin LAR 30 mg SC every month	
Simo et al. [28]	9/9	–	GTR (2), STR (7)	9/9	–	–	1/9	0/9	–	Sandostatin LAR IM every month; 30 mg for first two cycles and 40 mg thereafter	
Norden et al. [29]	33/34	–	–	28/34	–	–	–	13/34	–	Pasireotide LAR 60 mg IM every month	
Temozolomide											
Chamberlain. (2003)	16/16	–	GTR (4), STR (9), Biopsy (3)	12/16	–	54 Gy	–	–	–	75 mg/m^2^ PO temozolomide for 42 days followed by a 28-day break. 1 cycle = 10 weeks.	
Belanger (2022)	11/11	–	–	11/11	–	–	–	–	–	75 mg/m^2^ PO temozolomide	
PD-L1 blockers											
Bi et al. [34]	25/25	–	–	25/25	–	–	–	7/25	–	240 mg nivolumab IV biweekly.	
Brastianos et al. [35]	25/25	2.5	–	24/25	–	–	–	10/25	Bevacizumab (2), carboplatin (1), etoposide (1), GSK2256098 (1), SSAs (1), temozolomide (1)	200 mg pembrolizumab IV every three weeks	
Tyrosine kinaseinhibitors											
Horak et al. [22]	–	–	–	–	–	–	–	–	–	400 mg imatinib OD	
Raizer et al. [27]	24/24	2	–	18/24	1	–	12/24	10/24	Hydroxyurea (9), imatinib (3), mifepristone (1), temozolomide (4)	250 mg vatalanib BD increased by 250 mg/day every week until 750 mg BD reached	

*GTR* gross total resection, *STR* subtotal resection, *BD* twice daily, *Gy* Gray, *OD* once daily, *PO* per oral, *SR* sustained release, *SRS* stereotactic radiosurgery, *SSA* somatostatin analog, *TDS* three times daily

Systemic therapies were diverse and evaluated across varying clinical contexts, including differences in WHO grade distribution, prior therapies, and eligibility criteria. Agents included hydroxyurea (*n*= 8 studies) [13–16, 21, 23, 30, 32], bevacizumab (*n*= 5) [24, 25, 31, 33, 36], somatostatin analogs (SSAs, *n*= 5) [17, 19, 20, 28, 29],, temozolomide (*n*= 2) [37, 38], interferon-α (*n*= 3) [12, 18, 26], PD-1/PD-L1 inhibitors (*n*= 2) [34, 35], and tyrosine kinase inhibitors (TKIs, *n*= 2) [22, 27]. Two studies evaluated combination regimens (Tables 1and 2) [24, 30, 31].

### Progression free survival

#### WHO grades 1, 2, and 3

**Fig. 2:**
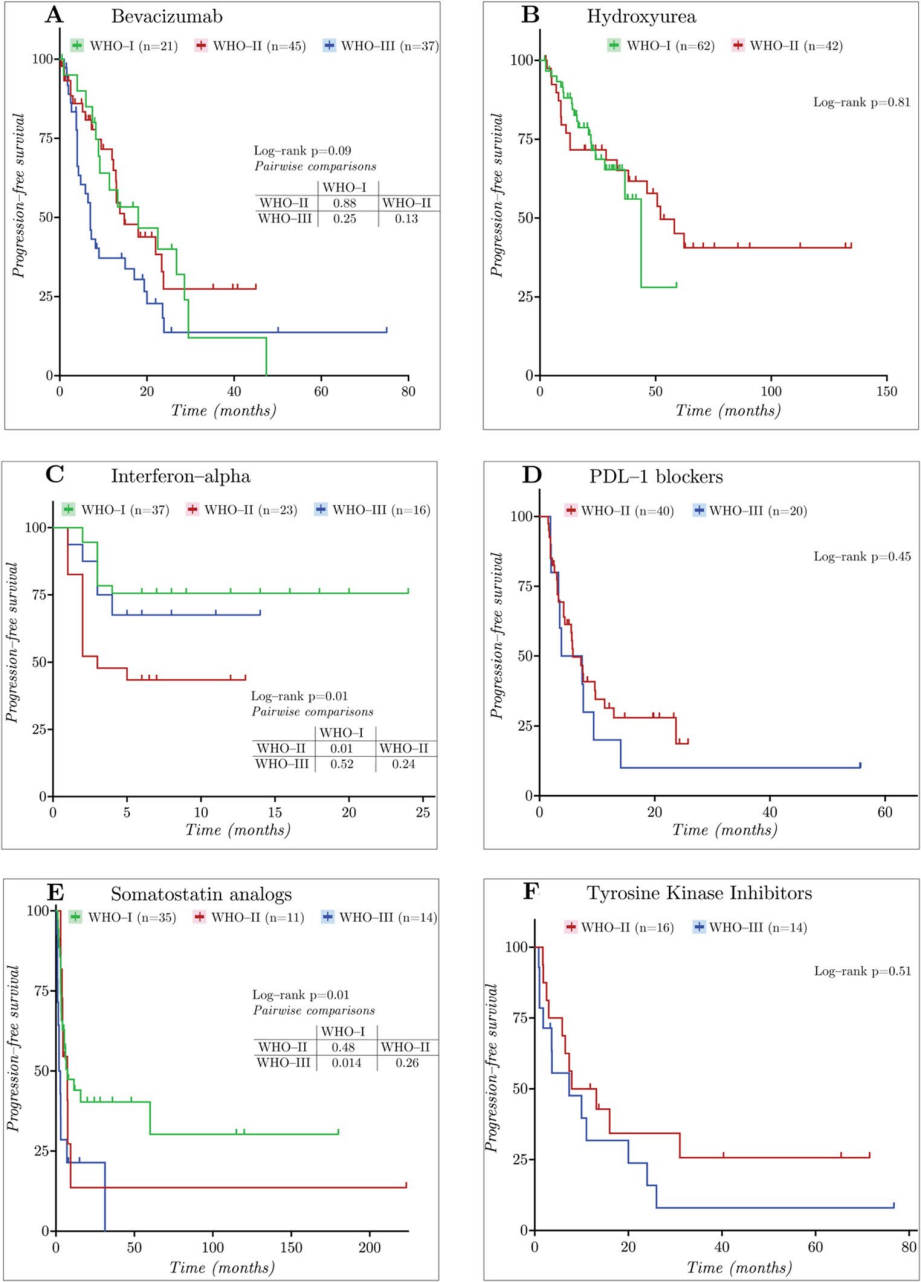
Kaplan–Meier PFS survival curves for WHO-I, WHO-II, and WHO-III tumors

Bevacizumab, interferon-α, and SSAs had adequate data for full grade-level comparisons (Fig. 2; Table 3). Due to insufficient data, analyses were limited to grades 1 and 2 for hydroxyurea, and grades 2 and 3 for PD-1/PD-L1 inhibitors and tyrosine kinase inhibitors (TKIs).

**Table 3:** Characteristics of included systemic agents as frequencies (%) and medians (range)

Variable		Drug						
	Overall	Bevacizumab	HU	INF-α	PDL-1 Blocker	SST analog	TKI	Temozolomide
No. of studies	27	5	8	3	2	5	2	2
Patients	511	103 (20.2%)	144 (28.2%)	76 (14.9%)	50 (9.8%)	78 (15.3%)	33 (6.5%)	27 (5.3%)
Sex								
Male	190 (37.2%)	53 (51.5%)	47 (32.6%)	17 (22.4%)	9 (36%)	39 (50%)	16 (48.5%)	9 (33.3%)
Female	296 (57.9%)	50 (48.5%)	97 (%)	59 (77.6%)	16 (64%)	39 (50%)	17 (51.5%)	18 (66.7%)
Not reported	25	0	0	0	25	0	0	0
Age, range	60 (20–89)	57 (20–89)	60 (26–86)	63 (36–88)	–	54 (21–87)	59 (30–88)	57 (22–82)
Functional status								
KPS	80 (50–100)	80 (60–100)	80 (50–100)	–	80 (70–100)	80 (50–100)	80 (60–100)	–
ECOG	1 (0–3)	1 (0–3)	1.5 (1–3)	–	0 (0–1)	–	–	–
WHO Grade								
I	158 (32.6%)	21 (20.4%)	62 (43.1%)	37 (48.7%)	0 (0.0%)	35 (44.9%)	3 (9.1%)	18 (66.7%)
II	177 (36.6%)	45 (43.7%)	42 (29.2%)	23 (30.3%)	40 (80.0%)	11 (14.1%)	16 (48.5%)	7 (25.9%)
III	92 (19.0%)	37 (35.9%)	1 (0.01%)	16 (21.1%)	10 (20.0%)	14 (17.9%)	14 (42.4%)	2 (7.4%)
II/III	57 (11.8%)	0 (0%)	39 (27.1%)	0 (0%)	0 (0.0%)	18 (23.1%)	0 (0.0%)	0 (0.0%)
Site								
Convexity	128 (50.0%)	–	62 (45.3%)	45 (64.3%)	15 (60.0%)	6 (25.0%)	–	12 (44.4%)
Skull base	102 (39.8%)	–	61 (44.5%)	19 (27.1%)	8 (32.0%)	14 (58.3%)	–	4 (14.8%)
Multiple/mixed	26 (10.2%)	–	14 (10.2%)	6 (8.6%)	2 (8.0%)	4 (16.7%)	–	–
Not reported	228	103	7	6	25	54	33	11 (40.7%)
Primary surgical resection								
GTR	69 (31.4%)	13 (31.0%)	25 (30.1%)	24 (33.3%)	–	7 (30.4%)	–	4 (25.0%)
STR	135 (61.4%)	29 (69.0%)	53 (63.9%)	39 (54.2%)	–	14 (60.9%)	–	9 (56.3%)
Biopsy	16 (7.3%)	0 (0.0%)	5 (6.0%)	9 (12.5%)	–	2 (8.7%)	–	3 (18.8%)
Not reported	264	61	61	4	50	55	33	11
Prior surgery count	2 (0–10)	2 (1–10)	1 (1–7)	2 (1–4)	–	2 (0–5)	2 (1–6)	–
Prior radiation								
Yes	297 (66.9%)	73 (74.5%)	79 (55.2%)	47 (61.8%)	25 (100.0%)	55 (70.5%)	18 (75.0%)	23
No	147 (33.1%)	25 (25.5%)	64 (44.8%)	29 (38.2%)	0 (0.0%)	23 (29.5%)	6 (25.0%)	4
Not reported	40	5	1	0	25	0	9	
Prior radiation count	1 (0–4)	1.5 (1–4)	–	–	–	–	1 (0–2)	–
Prior SRS								
Yes	163 (50.6%)	41 (39.8%)	44 (50.6%)	58 (77.3%)	–	8 (24.2%)	12 (50%)	–
No	159 (49.4%)	62 (60.2%)	43 (49.4%)	17 (22.7%)	–	25 (75.8%)	12 (50%)	–
Not reported	162	0	57	1	50	45	9	27
Prior chemotherapy								
Yes	151 (48.2%)	32 (31.1%)	2 (28.6%)	72 (94.7%)	7 (28.0%)	28 (35.9%)	10 (41.7%)	–
No	162 (51.8%)	71 (68.9%)	5 (71.4%)	4 (5.3%)	18 (72.0%)	50 (64.1%)	14 (58.3%)	16 (100%)
Not reported	171	0	137				9	11
No. of cycles	4 (0.5–37)	4 (1–23)	5 (0.5–37)	4 (1–20)	–	5 (1–15)	2.8 (0.9–22)	2 (1–2)

Median (range) and percentages are calculated using only patients for whom data were reported; ‘not reported’ categories were excluded from these calculations. *ECOG* Eastern Cooperative Oncology Group,* GTR* gross total resection, *HU* hydroxyurea, *INF-α* interferon alpha, *KPS* Karnofsky Performance Scale, *PDL-1* programmed death ligand-1, *SRS* stereotactic radiosurgery, *SST* somatostatin, *STR* subtotal resection, *TKI* tyrosine kinase inhibitor, *TMZ*, temozolomide, *WHO* World Health Organization

Median PFS ranged from 7.4 to 43.8 months for grade 1, 3.0 to 64.8 months for grade 2, and 2.4 to 10.2 months for grade 3 tumors. In global grade-based comparisons, interferon-α (*p* = 0.01) and SSAs (*p* = 0.01) demonstrated significant differences in PFS across grades, while bevacizumab did not (*p* = 0.09). In pairwise analyses, interferon-α had a longer PFS in grade 1 compared with grade 2 (*p* = 0.01), with no significant difference between grades 2 and 3. For SSAs, PFS was longer in grade 1 than grade 3 tumors (7.4 vs. 2.4 months, *p* = 0.014), with no other significant pairwise differences. Bevacizumab showed no significant PFS differences across grades. In grade-restricted analyses, median PFS was comparable for hydroxyurea (grade 1 vs. 2, *p* = 0.81), PD-L1 inhibitors (grade 2 vs. grade 3, *p* = 0.45), and TKIs (grade 2 vs. 3, *p* = 0.51) (Figure S1A).

Landmark analyses demonstrated early PFS separation with interferon-α, with higher PFS-6 in grade 1 compared with grade 2 tumors (*p* = 0.040). A nominally significant difference was observed for bevacizumab at the 12-month landmark, favoring grade 2 over grade 3 (68.3% vs. 37.2%, *p* = 0.031); however, this isolated finding was not supported by a significant global log-rank comparison across grades (*p* = 0.09) and was not consistently observed at other landmarks. No additional agents demonstrated statistically significant landmark differences, although numerically higher PFS was generally observed in lower-grade tumors (Table 4).

**Table 4:** PFS-6, 12–, 18–, and 24 months

Drug/ WHO Grade	*n*	Median PFS (95% CI)	Progression-free survival (95% CI)			
			PFS–6	PFS–12	PFS–18	PFS–24
Bevacizumab						
I	21	18.0 (8.3–28.6)	85.0% (60.4–94.9)	58.7% (34.0–76.8)	46.7% (23.3–67.1)	40.0% (17.8–61.5)
II		14.8 (12.3–23.8)	80.8% (65.1–89.9)	68.3% (50.5–80.8)	43.8% (26.2–60.2)	27.4% (11.4–46.2)
III		7.0 (4.0–15.0)	57.5% (39.7–71.8)	37.2% (21.6–52.8)	30.4% (16.0–46.2)	13.7% (3.9–29.5)
II/III	82	13 (7.3–18)	69.9% (58.2–78.9)	53.4% (41.2–64.2)	37.8% (26.0–49.5)	20.7% (10.6–33.1)
Hydroxyurea						
I	63	43.8 (28.0–NR)	95.0% (85.2–98.3)	88.1% (76.7–94.2)	78.7% (65.4–87.3)	68.6% (53.4–79.8)
II	42	52.1 (33.3–NR)	92.4% (78.2–97.5)	77.0% (60.4–87.3)	71.7% (54.7–83.2)	–
II/III	82	33.3 (9–52.1)	68.4% (56.8–77.5)	57.9% (45.4–68.6)	52.5% (39.7–63.7)	–
INF-α						
I	37	NR	75.6% (58.3–86.5)	–	–	–
II	23	3 (2–NR)	43.5% (23.3–62.1)	–	NA	NA
III	16	NR	67.5% (38.4–85.1)	–	NA	NA
II/III	39	NR	52.9% (35.9–67.3)	–	NA	NA
SST analog						
I	35	7.4 (3.5–NR)	56.9% (38.9–71.3)	44.0% (26.9–59.8)	40.3% (23.6–56.5)	–
II	11	7.3 (3.1–9.4)	54.5% (22.9–78.0)	13.6% (0.8–44.3)	–	–
III	14	2.4 (1.0–7.0)	28.6% (8.8–52.4)	21.4% (5.2–44.8)	–	–
II/III	43	3.1 (2.0–3.9)	27.9% (15.6–41.6)	13.0% (4.6–26.0)	8.7% (2.0–21.9)	–
PD-L1 blockers		5.8 (4.2–9.4)				
II	39	5.8 (4.2–11.3)	46.7% (30.2–61.7)	29.2% (15.5–44.4)	22.7% (10.5–37.7)	8.1% (1.6–21.7)
III	11	5.6 (1.9–9.4)	54.5% (22.9–78.0)	27.3% (6.5–53.9)	13.6% (1.0–42.6)	–
II/III	50	5.8 (4.2–9.4)	48.7% (34.0–61.8)	28.8% (16.6–42.0)	20.9% (10.4–34.0)	9.4% (2.7–21.3)
TKIs						
II	16	10.5 (3.0–NR)	68.8% (40.5–85.6)	50.0% (24.5–71.0)	34.3% (12.2–58.0)	–
III	14	7.4 (1.0–20)	55.6% (26.4–77.2)	31.7% (9.9–56.5)	–	15.9% (2.6–39.7)
II/III	30	7.9 (3.7–20)	62.9 (43–77.5)	41.9 (24.1–58.8)	33.9 (17.3–51.2)	25.4 (10.9–42.8)
Temozolomide						
I	18	NR	83.3% (56.8–94.3)	–	–	–

“–” represents no additional progression events occurred during that interval, while “NA” indicates that no patients remained at risk at that time point, precluding estimation of survival probability. NR, not reached

#### WHO grades I and II/III

**Fig. 3:**
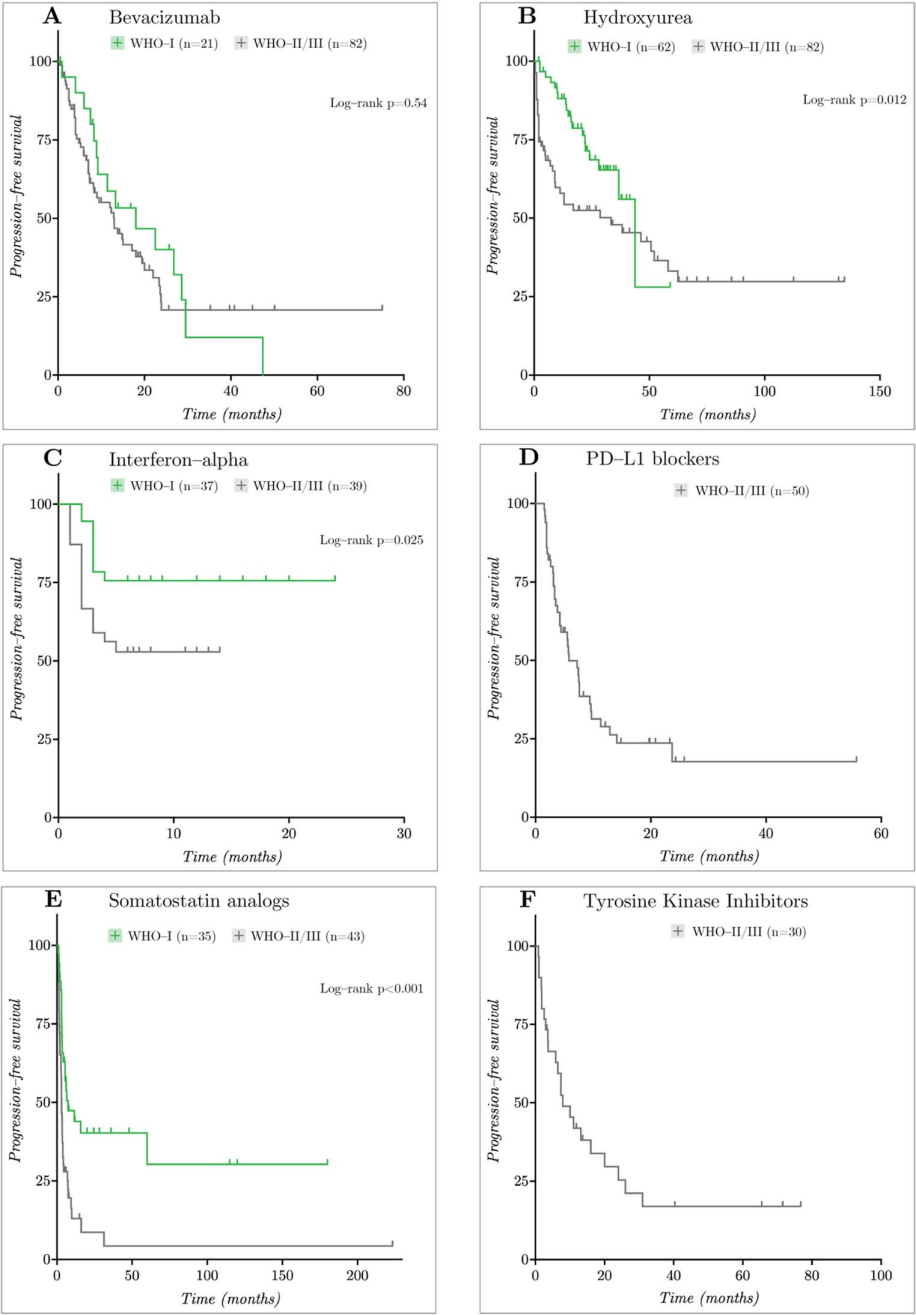
Kaplan–Meier PFS survival curves for WHO-I and WHO-II/III tumors

Median PFS was consistently longer in grade 1 tumors compared to grade 2/3 across all agents (Fig. 3; Table 4). In global log-rank testing, hydroxyurea (43.8 vs. 33.3 months, *p* = 0.01), interferon-α (*p* = 0.02), and SSAs (7.4 vs. 3.1 months) demonstrated longer PFS in grade 1 tumors, while bevacizumab showed comparable results (18 vs. 13 months, *p* = 0.54).

Landmark analyses at 6, 12, 18, and 24 months supported these trends. Grade 1 tumors treated with hydroxyurea maintained higher PFS-6 (95.0% vs. 68.4%, *p* = 0.0012), PFS-12 (88.1% vs. 57.9%, *p* < 0.001) and PFS-18 (78.7% vs. 52.5%, *p* = 0.0048), with later landmarks limited by small risk sets. SSAs also showed higher PFS-6 (56.9% vs. 27.9%, *p* = 0.012), PFS-12 (44.0% vs. 13.0%, *p* = 0.0044), and PFS-18 (40.3% vs. 8.7%, *p* = 0.0035) in grade 1 tumors. For interferon-α, grade 1 tumors had higher PFS-6 than grade 2/3 tumors (75.6% vs. 52.9%, *p* = 0.047). In contrast bevacizumab showed numerically higher landmark PFS rates in grade 1 tumors, but none of these differences reached statistical significance. Across all agents combined grades 2/3 tumors exhibited poorer PFS than grade 2 tumors alone, with exceptions observed for interferon- α (PFS-6: 52.9% vs. 43.5%), PD-1/PD-L1 blockers (PFS-6: 48.7% vs. 46.7%; PFS-12: 28.8% vs. 29.2%), and SSAs (PFS-12: 13.0% vs. 13.6%).

### Overall survival

Extractable OS data were available for 189/484 patients (39%), spanning five drugs across nine cohorts, whereas PFS data were available for all patients. Among grade 1 tumors, median OS was 32.3 months (95% CI: 19.7–36.1) with bevacizumab and 8 months (95% CI: 4–14) with interferon-α. Pairwise Compared to grade 1, OS comparisons for bevacizumab showed no statistically significant differences with grade 2 (26.9 months [95% CI: 20.5–NA], *p* = 0.58), grade 3 (19 months [95% CI: 9–25.6], *p* = 0.27), and grade 2/3 (23.9 months [95% CI: 18–NA], *p* = 0.69).

For grade 2 tumors, median OS was 25.8 months (95% CI: 17.6–7.9) with PD-1/PD-L1 inhibitors and 26.0 months (95% CI: 11.2–NA) for TKIs. For grade 2/3 tumors, median OS was 14.5 months (95% CI: 5.3–29.8) with somatostatin analogs, 31.1 months (95% CI: 17.6–NA) with PD-1/PD-L1 inhibitors, and 24.7 months (95% CI: 11.8–42.1) with TKIs. Supplementary Figure S1B non-comparatively summarizes median OS, with pooled landmark OS rates presented in Table 5.

**Table 5:** OS-6, 12–, 18–, and 24 months

Drug/ WHO Grade	*n*	Median OS (95% CI)	Overall survival (95% CI)			
			OS–6	OS–12	OS–18	OS–24
Bevacizumab						
I	14	32.3 (19.7–36.1)	92.9% (59.1–99.0)	92.9% (59.1–99.0)	85.7% (53.9–96.2)	77.9% (45.9–92.3)
II	31	26.9 (20.5–NR)	93.4% (76.2–98.3)	86.0% (66.7–94.5)	73.8% (52.5–86.7)	64.3% (41.9–79.8)
III	24	19.0 (9.0–25.6)	83.3% (61.5–93.4)	65.9% (43.1–81.3)	50.2% (27.8–69.0)	32.5% (13.3–53.5)
II/III	55	23.9 (18.0–NR)	88.8% (76.7–94.8)	76.7% (62.6–86.1)	63.0% (47.7–75.0)	50.0% (34.3–63.7)
INF-α						
I	37	8 (8–14)	59.2% (41.7–73.)	34.3% (19.1–50.1)	15.6% (5.7–29.8)	6.2% (0.6–21.7)
SST analog						
II/III	17	14.5 (5.3–29.8)	76.0% (48.0–90.3)	53.2% (25.5–74.7)	44.3% (18.1–67.9)	33.3% (9.7–59.5)
PDL-1 Blockers						
II	18	25.8 (17.6–37.9)	88.9% (62.4–97.1)	83.3% (56.8–94.3)	65.5% (38.6–82.8)	52.4% (26.7–72.9)
II/III	25	31.1 (17.6–NR)	88.0% (67.3–96.0)	84.0% (62.8–93.7)	67.2% (44.9–82.1)	58.2% (36.2–75.0)
TKIs						
II	14	26.0 (11.2–NR)	85.7% (53.9–96.2)	64.3% (34.3–83.3)	57.1% (28.4–78)	50.0% (22.9–72.2)
II/III	22	24.7 (11.8–42.1)	86.4% (63.4–95.4)	68.2% (44.6–83.4)	59.1% (36.1–76.2)	50.0% (28.2–68.4)

*NR* not reached, *SST* somatostatin, *TKI* tyrosine kinase inhibitor

### Adverse effects

Hematologic toxicity was rare with bevacizumab, with pooled patient-level rates below 1% for both low-grade (1/2) and severe (≥ 3) events (Supplementary Tables S1 and S2). Treatment discontinuation occurred in 16.2% of patients (95% CI: 10.5–24.3; I²=0%), most commonly due to disease progression rather than toxicity; the largest prospective phase II cohort attributed discontinuation to progression in 42% and toxicity in 14% [36]. Contrastingly, 67.4% of patients experienced any hematologic event with hydroxyurea (95% CI: 26.3–92.3; I²=57.4%) across five studies (grade 1/2: 38.7%; grade ≥ 3: 11.7%). Non-hematologic toxicity (3.9%, all grade 1/2) and treatment discontinuation (3.0%) were uncommon. However, dose modifications and reasons for discontinuation were inconsistently reported, suggesting that pooled discontinuation rates likely underestimate treatment burden despite frequent cytopenias. Cycle-level analyses reinforced this, showing hematologic toxicity in 13.5% of cycles (predominantly low grade), severe events in 1.3%, and non-hematologic events in 17.1% of cycles (I²=93.7%).

Interferon-α was associated primarily with hematologic events in 11.9% of cycles (grade 1/2: 8.0%; grade ≥ 3: 3.6%). Non-hematologic toxicity occurred in 17.6% of cycles, with 3.6% reaching grade ≥ 3. Where CTCAE grade-specific data were available, severe non-hematologic events were predominantly fatigue, with rare infections and isolated events (e.g., constipation or thrombophlebitis). Discontinuation due to toxicity was uncommon (3.3%). Somatostatin analogs were associated with minimal hematologic toxicity (2.6%, all grade 1/2), but non-hematologic events were more frequent, affecting 48.3% of patients (95% CI: 20.6–77.0; I²=75%). Despite this, discontinuation rates remained low (4.1%). Non-hematologic events were mainly gastrointestinal (diarrhea, nausea/anorexia, abdominal pain/flatulence), with severe toxicity uncommon and largely driven by the pasireotide cohort (e.g., grade 3 pancreatic enzyme deviations and metabolic abnormalities) [29].

### Risk of bias assessment

Using the ROBINS-I tool, sixteen studies (59%) had a serious risk of bias and 11 (41%) a moderate risk (Supplementary Figure S2). Confounding (D1) was serious in 26 studies. Selection bias (D2) was serious in 2 studies, low in 3, and moderate in 22. Classification of interventions (D3) was predominantly low risk (23/27). Deviations from intended interventions (D4) were serious in 2, moderate in 5, and low in 20. Bias due to missing data (D5) was moderate in 6 and low in 21. Outcome measurement (D6) showed serious risk in 4, moderate in 21, and low in 2 studies. Selective reporting (D7) was serious in 3, moderate in 22, and low in 3. Given the non-randomized design of all included studies and the predominance of moderate-to-serious risk of bias, all estimates should be interpreted as descriptive benchmarks rather than causal treatment effects. Informal certainty ratings aligned with ROBINS-I were low for bevacizumab, PD-1/PD-L1 inhibitors, and somatostatin analogs, and very low for hydroxyurea, interferon- α, and TKIs.

## Discussion

This study presents the first IPD meta-analysis integrating reconstructed and reported survival data to investigate systemic therapies for recurrent or refractory meningiomas. Median PFS ranged from 7.4 to 43.8 months for WHO grade 1, 3.0–64.8 months for grade 2, and 2.4–10.2 months for grade 3. The highest reported median PFS values, particularly those exceeding 60 months in grade 2 tumors, were driven from very small cohorts with substantial right censoring and likely reflect limited event accrual than durable treatment benefit. Median OS across all agents ranged from 8 to 32 months, without grade-specific differences. Hydroxyurea had the highest rates of hematologic toxicity (67% any grade; 12% grade ≥ 3), whereas bevacizumab and somatostatin analogs were generally well tolerated, with discontinuation rates of 16% and 4%, respectively. Interferon-α was primarily associated with low-grade cytopenias, and non-hematologic toxicity remained modest across all classes. Together, these results establish the first harmonized benchmarks for efficacy and safety of systemic therapies in recurrent meningioma, highlighting limited objective activity but durable stabilization with acceptable tolerability in selected regimens.

Systemic therapies investigated for recurrent/refractory meningioma span cytotoxic, biologic, anti-angiogenic, receptor-kinase, and immunomodulatory classes (Supplementary Table 11) [17, 18, 23, 31, 34, 35, 39], with apparent differences between agents likely reflecting patient selection and surveillance practices rather than true therapeutic superiority.

### Cytotoxic and early biologic agents

#### Hydroxyurea

Hydroxyurea achieved prolonged disease stabilization, with median PFS of 44 months in grade 1 and 33 months in grade 2/3 disease, consistent with a predominantly cytostatic effect [40]. These estimates contextualize prior single-study signals, including the Karsy et al. phase I/II cohort where a high PFS-6 rate (85%) did not translate into durable control (median PFS, 8 months) [30]. For toxicity patterns, hematologic events were common (any-grade: 67%; grade ≥ 3: 12%), while non-hematologic events and discontinuations were infrequent, reflecting protocol-mandated dose modifications for cytopenias in several prospective studies and supporting a cytostatic treatment profile.

Prospective monotherapy studies are concordant, showing no radiographic regressions, stabilization in a subset, and clinically relevant myelosuppression with occasional withdrawal or transition to surgery [41]. Platelet-derived growth factor receptor (PDGFR) co-inhibition has been investigated with hydroxyurea, with a dual-center phase II trial reporting stable disease as the best response (overall PFS-6, 61.9%; grade 1, 87.5%; grade 2/3, 46.2%) and median PFS 7.0 months [42]. Additionally, a randomized dataset demonstrated superior outcomes with hydroxyurea monotherapy compared with combined hydroxyurea/imatinib (PFS-9, 75% vs. 0%; median PFS, 19.5 vs. 4.0 months; median OS, 27.5 vs. 6 months), suggesting PDGFR blockade may not enhance the cytostatic benefit of hydroxyurea [43].

####  Somatostatin analogs

SSA monotherapy produced a median PFS of 7.4 months in grade 1 vs. 3.1 months in grade2/3, with PFS-6 of 56.9% and 27.9%, respectively. Outcomes were more favorable in grade 1 tumors, consistent with a stabilization-dominant effect rather than cytoreduction, although grade dependence may reflect underlying biology, patient selection, or between-study differences, and should be considered hypothesis generating. These findings align with the pasireotide LAR phase II signal (PFS-6 50% in grade 1 vs. 17% in grade 2) and the generally low objective response rates across single-agent SSA trials [29]. Across included cohorts, receptor-based enrichment was inconsistent, with some studies requiring SSTR positivity on octreotide imaging and others not mandating or reporting screening. Notably, a subset in the pasireotide LAR trial demonstrated high tracer uptake [**Ref**]. Toxicity reporting and dosing schedules varied across studies, and agent- or dose-specific toxicity differences should therefore be interpreted descriptively.

Two prior IPD syntheses provide context but differ materially from our approach. One combined SSAs with SSTR-targeted peptide receptor radionucleotide therapy, reporting higher disease control and PFS that likely reflect PET-selected cohorts and the greater efficacy of radionuclide therapy, limiting comparability with SSA studies [44]. The second pooled SSA with or without everolimus, noting 58% stable disease and a dose-response signal but overall very low certainty grading due to heterogeneity, potential reverse causality, and inconsistent endpoints [45]. The phase II CEVOREM trial of everolimus/octreotide reduced tumor growth rate with modest PFS and added toxicity (e.g., hyperglycemia) [46]. Another comparative cohort suggested similar PFS for everolimus/octreotide and sunitinib, but with selection and sequencing biases [47]. Finally, a large retrospective series of Sandostatin LAR in 43 patients similarly reported disease stabilization rather than objective response [48].

#### Interferon–α

IFN-α achieved moderate disease control, with median PFS 8 months in grade 1 and transient benefit in higher-grade disease, reinforcing a stabilization-without-shrinkage profile. These align with prior prospective series demonstrating immune-mediated antiproliferative effects but limited durability [18, 26, 49]. In the largest grade 1 trial, heavily pretreated patients received 10 MU/m^2^every other day and achieved no objective responses, a PFS-6 of 54%, PFS-12 of 31%, median TTP of 7 months, and median OS of 8 months; fatigue and cytopenias were the principal toxicities, with treatment discontinuation in 9% [18]. Non-hematologic toxicity reporting was inconsistent across studies, and pooled estimates therefore reflect only cohorts with graded adverse effect data. In high-grade cohorts, stable disease was observed in 60% but median PFS was limited to 12 weeks, highlighting the short-lived benefit in anaplastic disease [26]. Although outcomes appeared more favorable in grade 1 tumors, this pattern likely reflects underlying biology and clinical selection and should be considered hypothesis-generating.

### Targeted and anti-angiogenic therapies

#### Bevacizumab

Bevacizumab achieved a pooled median PFS of 13 months and OS of 24 months in grade 2/3 meningioma, representing intermediate efficacy signal earlier monotherapy series (Nayak et al. [25] median PFS 6 months; PFS-6 44%) and combination therapy with everolimus (Shih et al. [31] median PFS 22 months; PFS-6 69%), consistent with biological additivity via concurrent mTORC1 inhibition [50].

Prior bevacizumab-focused meta-analyses reported heterogeneous PFS ranges (7–19 months; PFS-6 44–80%) and mean OS of 24 months but relied on aggregate study-level data [33, 51, 52]. A recent meta-analysis identified WHO grade, sex, prior resection, line of therapy, and treatment duration as moderators of outcome [52]. Additional evidence supports this interpretation: low-dose regimens preferentially improved radiation necrosis rather than viable tumor [53]; volumetric-kinetic analyses confirmed marked growth rate and edema reduction [54]; a retrospective high-grade series showed short-term survival benefit [55]; and volumetric criteria proved more sensitive than RANO for capturing bevacizumab-related growth deceleration [56].

#### Tyrosine kinase inhibitors (TKIs)

TKIs achieved a pooled median PFS of 7.9 months and OS of 25 months, closely mirroring the sunitinib phase II signal in heavily pretreated grade 2/3 meningiomas (PFS-6 42–44%, median PFS 5.2 months, median OS 24.6 months), where intratumoral hemorrhage and thrombotic microangiopathy were notable toxicities [39]. In contrast, imatinib monotherapy was ineffective despite near-universal PDGFR expression (PFS-6 29.4% overall; 0% in WHO grade 2/3; median PFS 2 months) [57]. Accordingly, the randomized GICNO trial demonstrated superior outcomes with hydroxyurea monotherapy compared with combined hydroxyurea/imatinib (PFS-9 75% vs. 0%; median PFS 19.5 vs. 4.0 months), arguing against PDGFR-targeted combinations [58]. Modest TKI efficacy appears driven by VEGFR-directed multitarget inhibition (e.g., sunitinib) rather than PDGFR-specific blockade, consistent with reported correlations between VEGFR2/PDGFRβ expression and outcome [47].

###  Immune checkpoint inhibitors

PD-1/PD-L1 inhibitors (pembrolizumab, nivolumab) achieved a pooled PFS-6 of 46.7% and median PFS of 5.8 months, with median OS 25–31 months. A recent systematic review similarly characterized checkpoint blockade as modestly effective in recurrent grade 2/3 meningioma, with greater activity observed in mismatch repair (MMR)-deficient or hypermutated tumors [59]. Additional phase II data, including pembrolizumab cohorts co-enrolled with solitary tissue sarcoma failed to meet PFS-6 overall but documented isolated responses in atypical meningioma, supporting a biomarker-defined benefit [60]. Case-level reports and small biomarker studies suggest greater benefit in MMR–deficient or hypermutated tumors [60, 61].

###  Clinical implications and pragmatic framework

Consistent with international guidance, systemic therapy is generally reserved for progressive meningioma when further surgery or salvage radiotherapy is no longer feasible, with enrolment in prospective trials remaining the preferred approach [34181733]. When trial access is limited, our benchmark estimates support a pragmatic, patient- and grade-informed approach rather than assumptions of cross-agent superiority. For grade 1 (and select indolent grade 2) disease with SSTR expression, SSAs may be considered when a low-toxicity stabilization strategy is desired. For patients with prominent peritumoral edema or suspected radiation necrosis, bevacizumab may be appropriate given its anti-permeability effects and guideline inclusion. For more aggressive grade 2/3 disease or rapid progression after exhausted local therapy, VEGF/VEGFR-directed strategies (e.g., bevacizumab, sunitinib, or combined bevacizumab/everolimus) should be selected based on toxicity profiles and comorbidities. Immune checkpoint inhibitors remain investigational overall but may be considered in biomarker-defined subgroups (e.g., hypermutated/MMR-deficient tumors), preferentially within clinical trials [35402234].

### Strengths, limitations, and future directions

This meta-analysis captures most eligible published cohorts through a comprehensive search and dual screening process. Strengths include integration of reconstructed time-to-event data, standardized toxicity grading, and Kaplan–Meier–based censoring, mitigating reporting bias across heterogeneous studies. Several limitations warrant consideration. Most studies were non-randomized, single-arm cohorts with heterogeneous patient selection and variability in prior interventions. The absence of harmonized tumor volume or growth-rate kinetics and molecular subclassification limits risk adjustment and likely contribute to confounding by indication. Biomarker reporting was inconsistent and rarely linked to patient-level outcomes, precluding biomarker-stratified pooled analyses. Imaging response criteria (RANO, RECIST, Macdonald, or unspecified) varied considerably, introducing classification bias, while some analyses relied on reconstructed rather than original survival curves. Non-uniform progression definitions and imaging intervals may have biased PFS estimates, as longer surveillance intervals can artifactually prolong PFS, whereas more frequent surveillance may shorten it through earlier detection. OS analyses were underpowered relative to PFS due to substantial missing data (61%) and the confounding influence of post-progression therapies. Finally, ROBINS-I identified a serious risk of bias due to confounding (D1) in 24 of 25 studies, reflecting non-random treatment allocation and substantial heterogeneity in eligibility criteria, grade mix, and prior therapy burden. Performance status, tumor volume or growth kinetics, and extent or timing of prior local treatments were inconsistently reported and could not be adequately adjusted for. Consequently, observed differences across agents may partly reflect baseline risk and treatment selection rather than true differential drug activity.

Nevertheless, reported benchmarks represent the most comprehensive synthesis of systemic therapy outcomes in recurrent meningioma, providing a practical reference for clinical counseling and trial design. Prospective, standardized, and biomarker-enriched studies are needed to validate findings and identify predictive subgroups, with systemic agents reserved for appropriately selected patients with refractory disease in the interim.

## Conclusion

This first comprehensive IPD meta-analysis of systemic chemotherapy and targeted agents in recurrent or refractory intracranial meningioma harmonizes reconstructed survival data across heterogeneous studies to provide the most robust pooled benchmarks to date for efficacy, safety, and class-specific activity. Across therapeutic classes, systemic agents demonstrate predominantly cytostatic effects, with disease stabilization rather than radiographic regression as the main outcome.

## Supplementary Information

Below is the link to the electronic supplementary material.

Supplementary Material 1 (DOCX 940 KB)

## Data Availability

All extracted study-level data and reconstructed de-identified time-to-event dataset analyzed in this study will be made available from the corresponding author upon reasonable request.
