# Supplementary material for: Systemic therapy in the treatment of recurrent or refractory intracranial meningiomas: A systematic review and individual patient data meta-analysis
Source: Neurosurg Rev. 2026 Apr 16;49(1):353. doi: 10.1007/s10143-026-04233-w (PMC13086805; doi:10.1007/s10143-026-04233-w)
Supplement: Supplementary file 1 — Supplementary Material 1 (DOCX 940 KB) [file 10143_2026_4233_MOESM1_ESM.docx]

**Supplementary Appendix**

**Table of Contents**

|  |  |  |
| --- | --- | --- |
| **Methods S1** | Study design and registration | Page 2 |
|  |  |  |
|  |  |  |
| **Methods S2** | Plot Digitization Workflow | Page 2 |
|  |  |  |
|  |  |  |
| **Methods S3** | Data extraction | Page 2–3 |
|  |  |  |
| **Methods S4** | Statistical analysis | Page 3–5 |
|  |  |  |
|  |  |  |
| **FIGURE S1** | Median PFS and OS across different drugs/drug classes by WHO tumor grade. | Page 6 |
|  |  |  |
|  |  |  |
| **FIGURE S2** | Risk of Bias using the ROBINS-I Tool | Page 7 |
|  |  |  |
|  |  |  |
| **TABLE S1** | Reported hematologic and non-hematologic toxicities, stratified by grade and whether measured per patient or per treatment cycle, across included studies. | Pages 8–9 |
|  |  |  |
|  |  |  |
| **TABLE S2** | Toxicity grading harmonization across systems | Page 10 |
|  |  |  |
|  |  |  |
| **TABLE S3** | Pooled estimates of hematologic and non-hematologic toxicities, summarized by drug class, presented as proportions with 95% confidence intervals. | Page 11 |
|  |  |  |
|  |  |  |
| **TABLE S4** | Pharmacological mechanisms of action of drugs used in study. | Page 12 |
|  |  |  |
|  | |  |
| **Appendix References** | | Pages 13–15 |
|  |  |  |

**Methods S1: Study design and registration**

This systematic review was prospectively registered with PROSPERO (ID: CRD42025602986). ﻿The prespecified primary outcome was PFS, with planned subgroup analyses stratified by WHO tumor grade and systemic agent class. Secondary outcomes included OS and treatment-related toxicity. No substantive deviations from the registered protocol were made.

**Methods S2: Plot digitization workflow**

KM curves were digitized utilizing WebPlotDigitizer v4.0,^1^ and individual event times were reconstructed by combining digitized survival probabilities with reported numbers-at-risk and total event counts, following the approach described by Guyot et al.^2^ Two independent reviewers (EM and AG) performed digitization and reconstruction for each eligible curve. Reconstructed survival data were validated against reported study-level metrics, including median PFS/OS and landmark survival estimates when available. Curves were re-digitized and recalibrated to reported risk tables if reconstructed estimates differed from published values. Reconstructed event times represent pseudo-IPD extracted from published curves. Tied event times were expected and handled using standard KM and Cox model implementations. Interval censoring was not explicitly modeled, as original studies typically reported event status only at scheduled imaging assessments. When numbers-at-risk were incompletely reported, reconstruction relied on the closest available risk table or total event counts. Studies for which adequate reconstruction was not possible were excluded from quantitative pooling.

**Supplementary Methods S3: Data Extraction**

In retrospective cohorts, systemic therapy was typically administered as clinician-selected salvage in patients with radiographically progressive disease after prior local therapy (surgery and/or radiotherapy) when further local intervention was not feasible or was deferred; however, explicit rationale for selecting a specific agent (e.g., institutional preference, availability, receptor/biomarker status, comorbidity constraints, or prior drug exposure) was inconsistently reported, precluding harmonized adjustment for treatment line or selection criteria. Accordingly, all pooled estimates are intended as agent-specific benchmarks; we did not perform between-agent statistical comparisons or ranking. Because nearly all included studies were single-arm (non-randomized), we pooled prospective phase II trials and retrospective series to improve precision of benchmark estimates. As a robustness check, we repeated key PFS benchmarks restricting to prospective cohorts for agents where sufficient data were available; results were similar.

Accordingly, prior treatment variables (prior RT/SRS/chemotherapy and number of prior courses) were summarized descriptively (Table 2) rather than used for stratified analyses, because definitions and completeness varied across studies and were strongly shaped by eligibility criteria, limiting interpretable adjustment. Tumor location was summarized descriptively but not modeled as an effect modifier because it was missing in ~47% of patients (228/484) and location categories were variably defined across studies, limiting interpretable stratified analyses.

Functional status (KPS/ECOG) was reported in only a subset of studies, and KPS and ECOG were not available concurrently, limiting standardized adjustment. As an exploratory robustness check, we repeated analyses in the subset with reported performance status using a harmonized indicator (KPS ≥80 or ECOG 0–1), and grade effects were similar.

**Methods S4: Statistical analysis**

*S4.1 Radiographic progression criteria*

Across the 25 included cohorts, radiographic progression was assessed using Macdonald/modified Macdonald criteria in 11 studies, RANO criteria in 5, and RECIST in 1; the remaining 8 studies did not specify a formal response framework and instead defined progression based on investigator-assessed change on serial imaging (MRI/CT, and in one cohort with adjunct thallium-201 SPECT) (Table 1). Imaging surveillance schedules were also heterogeneous (often every 2–3 months when reported), so pooled PFS reflects each study’s operational definition and assessment cadence rather than a standardized RANO-based endpoint. Imaging intervals were not standardized across studies; therefore, PFS was treated as study-defined, and we emphasized pre-specified landmark estimates (6, 12, 18, and 24 months) commonly reported in recurrent meningioma trials and prior systematic reviews. In pairwise comparisons at each timepoint, multiplicity correction (Holm) was applied within each agent/drug class and timepoint for the set of pairwise grade comparisons performed at that landmark. Furthermore, comparisons of PFS and OS were done at each landmark timepoint compared to baseline. For additional interpretability and to incorporate studies reporting combined high-grade cohorts, grades 2 and 3 were collapsed (grade 2/3) and compared against grade I. This analysis was pre-specified to increase stability where grade 3 numbers were sparse and to enable inclusion of studies that reported only combined 2/3 outcomes. Where sample size permitted, sensitivity analyses maintaining grades 2 and 3 separately were performed. We did not plan formal quantitative comparisons between our pooled benchmarks and external series; where external figures are cited in the Discussion, they are provided for context only and are drawn preferentially from the largest/most relevant prospective studies and prior systematic syntheses to minimize selective citation. Prior treatment burden was summarized descriptively (Table 2).

*S4.2. Survival distributions*

Survival distributions were estimated using the Kaplan–Meier method with log–log transformed 95% confidence intervals (CIs). Median survival was summarized overall and stratified by WHO grade. Inferential comparisons between grades were performed using log-rank tests descriptively and estimated hazard ratios (HRs) using Cox proportional hazards models. Due to datasets pooling multiple cohorts per agent, primary inference used study-clustered robust standard errors to account for within-study correlation. Cox models were not used to support causal comparisons between different drugs or drug classes given strong confounding by indication and heterogeneous eligibility criteria across studies.

Proportional hazards assumptions were assessed using Schoenfeld residual tests and visual inspection of log-log survival plots. When proportional hazards were not supported, we emphasized nonparametric KM summaries and landmark estimates. Covariate adjustment was limited because patient-level prognostic factors (e.g., performance status, tumor volume, and prior treatment burden) were inconsistently reported across studies. Therefore, Cox models were primarily univariable with grade as the predictor.

*S4.3. Toxicity*

Toxicity outcomes were summarized using two distinct denominators: per patient (proportion of patients experiencing an event) and per treatment cycle (proportion of cycles with an event) when available **(Supplementary Table S1**). Because studies used different toxicity grading systems, including Common Terminology Criteria for Adverse Events (CTCAE) versions 1–5 and historical WHO criteria, toxicity grades were harmonized using threshold-based mapping of laboratory cutoffs where equivalence was demonstrable (e.g., hemoglobin, granulocyte, leukocyte, and platelet cutoffs) (**Supplementary Table S2**). For pooled proportions, we performed single-arm random-effects meta-analyses using a generalized linear mixed model (GLMM) with logit transformation. Zero-event studies were retained under the GLMM framework without ad hoc continuity corrections.

**Supplementary Figure S1.** Median PFS and OS across different drugs/drug classes by WHO tumor grade.


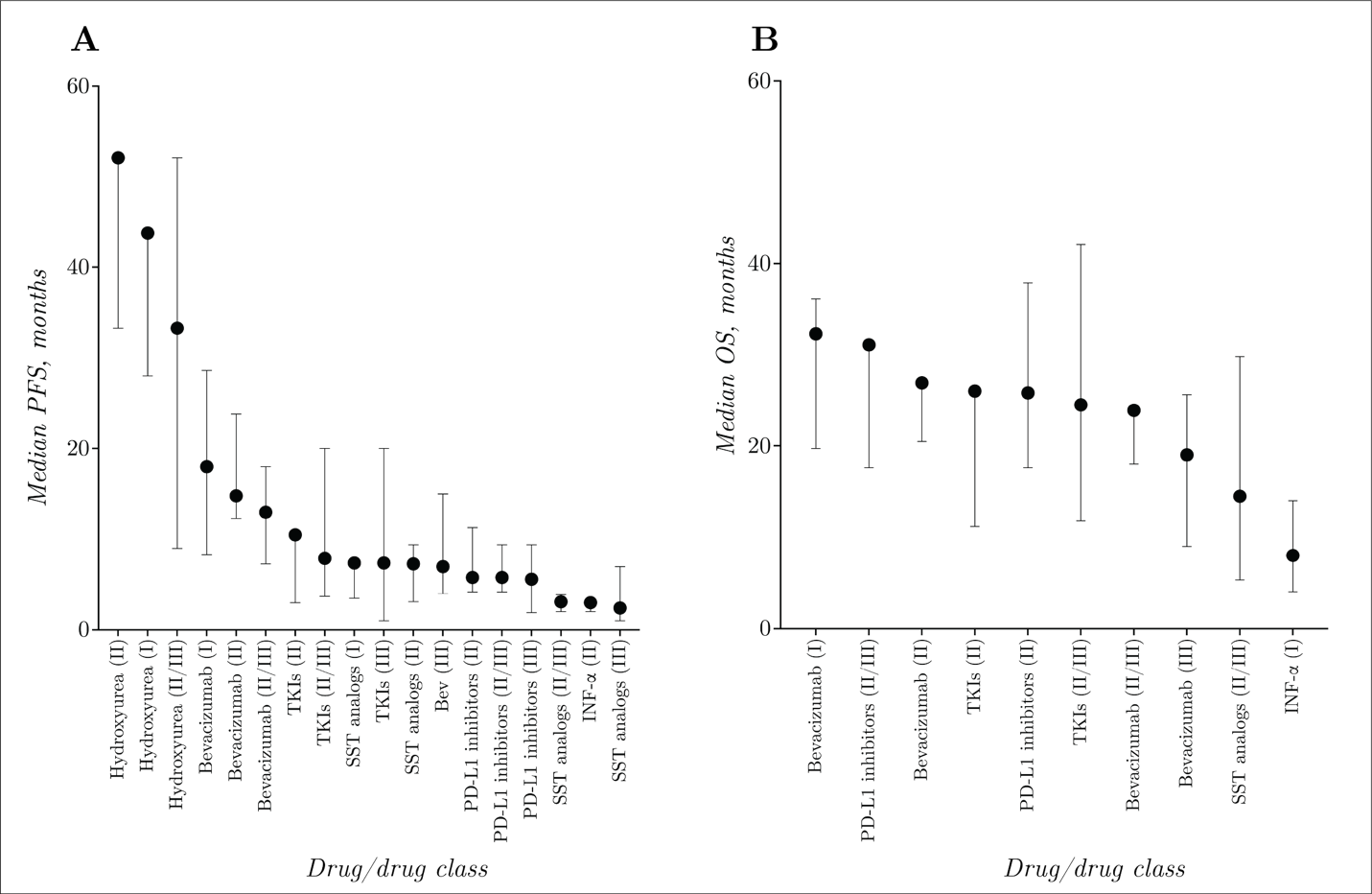


**Supplementary Figure S2.** Risk of Bias in Non-randomized Studies (ROBINS-I).


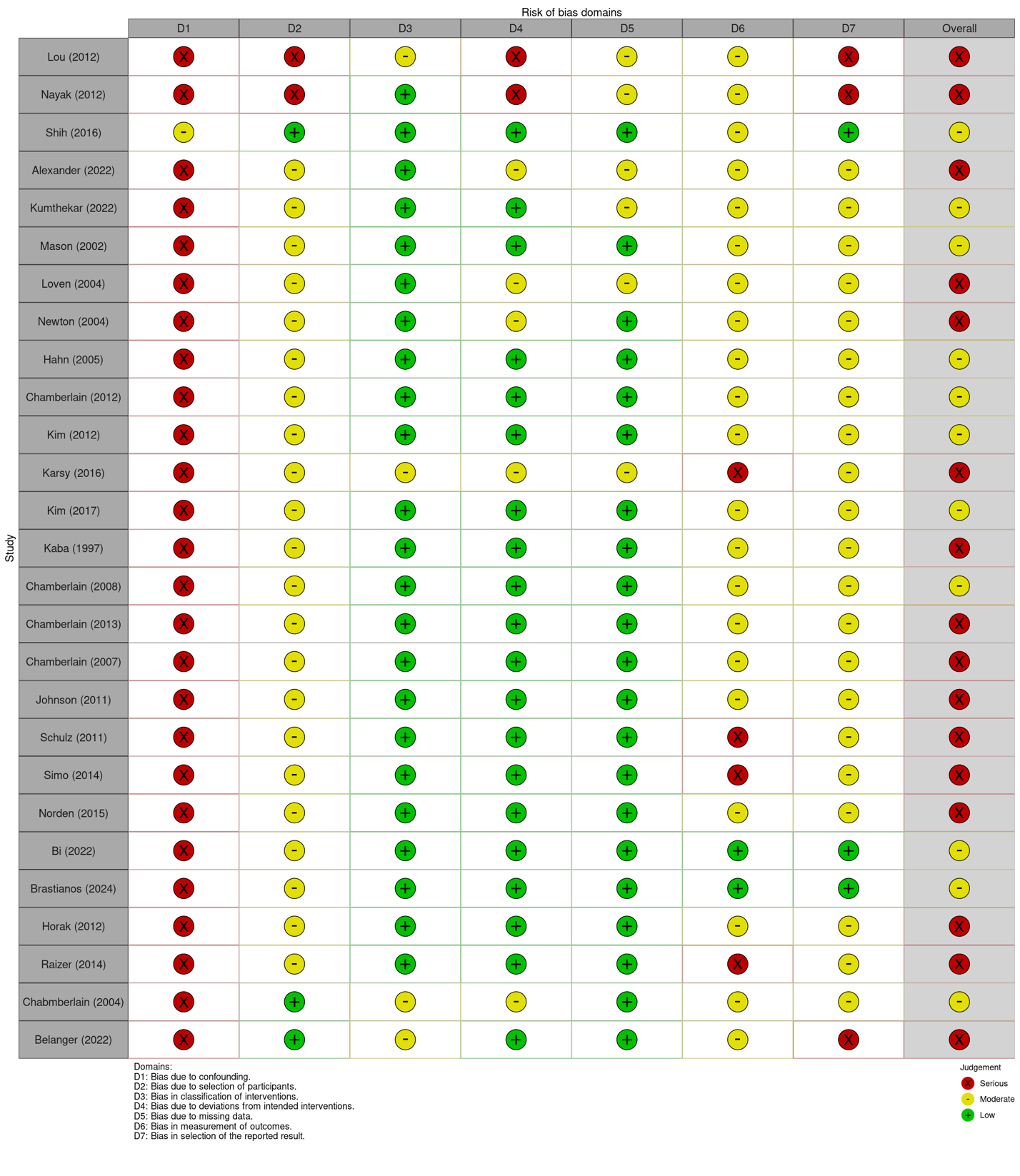


**Supplementary Table S1.** Hematologic and non-hematologic toxicities, stratified by grade and whether measured per patient or per treatment cycle.

|  |  |  |  |  |  |  |  |  | | |  |  |
| --- | --- | --- | --- | --- | --- | --- | --- | --- | --- | --- | --- | --- |
| **Study (Year)** | **Toxic criteria** | **Level reported** |  | **Hematologic toxicity** | | |  | **Non-hematologic toxicity** | | |  | **Discontinued treatment due to toxicity** |
|  |  |  |  |  |  |  |  |  | | |  |  |
|  |  |  |  | **Any toxicity** | **Grades I–II** | **Grades ≥3** |  | **Any toxicity** | **Grades I–II** | **Grades ≥3** |  |  |
|  |  |  |  |  |  |  |  |  |  |  |  |  |
|  |  |  |  |  |  |  |  |  |  |  |  |  |
| **Bevacizumab** |  |  |  |  |  |  |  |  |  |  |  |  |
| Lou (2012)^3^ | CTCAE 4.0 | Patient/ cycle |  | 2/14 | 1/14 | 1/14 |  | 18/NR | 17/NR | 1/NR |  | 2/14 |
|  |  |  |  |  |  |  |  |  |  |  |  |  |
|  |  |  |  |  |  |  |  |  |  |  |  |  |
| Nayak (2012)^4^ | NR | Patient |  | 0/15 | 0/15 | 0/15 |  | 3/15 | 3/15 | 0/15 |  | 3/15 |
|  |  |  |  |  |  |  |  |  |  |  |  |  |
|  |  |  |  |  |  |  |  |  |  |  |  |  |
| Shih (2016)^5^ | CTCAE 3.0 | Cycle |  | 20/NR | 19/NR | 1/NR |  | 87/NR | 78/NR | 9/NR |  | 4/17 |
|  |  |  |  |  |  |  |  |  |  |  |  |  |
|  |  |  |  |  |  |  |  |  |  |  |  |  |
| Alexander (2022)^6^ | NR | Patient |  | 0/23 | 0/23 | 0/23 |  | 8/23 | – | – |  | 2/23 |
|  |  |  |  |  |  |  |  |  |  |  |  |  |
|  |  |  |  |  |  |  |  |  |  |  |  |  |
| Kumthekar (2022)^7^ | NR | Cycle |  | 0/203 | 0/203 | 0/203 |  | 58/203 | 40/203 | 18/203 |  | 7/42 |
|  |  |  |  |  |  |  |  |  |  |  |  |  |
|  |  |  |  |  |  |  |  |  |  |  |  |  |
| **Hydroxyurea** |  |  |  |  |  |  |  |  |  |  |  |  |
| Mason (2002)^8^ | CTCAE 2.0 | Patient |  | 8/20 | 5/20 | 3/20 |  | 1/20 | 1/20 | 0/20 |  | 1/20 |
|  |  |  |  |  |  |  |  |  |  |  |  |  |
|  |  |  |  |  |  |  |  |  |  |  |  |  |
| Loven (2004)^9^ | WHO | Patient |  | 12/12 | 8/12 | 4/12 |  | 1/12 | 1/12 | 0/12 |  | 2/12 |
|  |  |  |  |  |  |  |  |  |  |  |  |  |
|  |  |  |  |  |  |  |  |  |  |  |  |  |
| Newton (2004)^10^ | NCI | Patient |  | 16/21 | – | – |  | 3/21 | – | – |  | 0/21 |
|  |  |  |  |  |  |  |  |  |  |  |  |  |
|  |  |  |  |  |  |  |  |  |  |  |  |  |
| Hahn (2005)^11^ | NCI | Patient |  | 9/14 | 9/14 | 0/14 |  | – | – | – |  | 1/14 |
|  |  |  |  |  |  |  |  |  |  |  |  |  |
|  |  |  |  |  |  |  |  |  |  |  |  |  |
| Chamberlain (2012)^12^ | NIH | Cycle |  | 13/88.5 | 12/88.5 | 1/88.5 |  | 24/88.5 | 22/88.5 | 2/88/5 |  | 0/35 |
|  |  |  |  |  |  |  |  |  |  |  |  |  |
|  |  |  |  |  |  |  |  |  |  |  |  |  |
| Kim (2012)^13^ | CTCAE 4.0 | Patient |  | 2/13 | 2/13 | 0/13 |  | 0/13 | 0/13 | 0/13 |  | 0/13 |
|  |  |  |  |  |  |  |  |  |  |  |  |  |
|  |  |  |  |  |  |  |  |  |  |  |  |  |
| Karsy (2016)^14^ | CTCAE 3.0 | Patient |  | 6/7 | 2/7 | 4/7 |  | 0/7 | 0/7 | 0/7 |  | 1/7 |
|  |  |  |  |  |  |  |  |  |  |  |  |  |
|  |  |  |  |  |  |  |  |  |  |  |  |  |
| Kim (2017)^15^ | CTCAE 3.0 | Cycle |  | 82/617 | 74/617 | 8/617 |  | 69/617 | 63/617 | 6/617 |  | 0/24 |
|  |  |  |  |  |  |  |  |  |  |  |  |  |
|  |  |  |  |  |  |  |  |  |  |  |  |  |
| **INF-α** |  |  |  |  |  |  |  |  |  |  |  |  |
| Chamberlain (2008)^16^ | CTCAE 3.0 | Cycle |  | 26/242 | 19/242 | 17/242 |  | 39/242 | 32/242 | 7/242 |  | 3/35 |
|  |  |  |  |  |  |  |  |  |  |  |  |  |
|  |  |  |  |  |  |  |  |  |  |  |  |  |
| Chamberlain (2013) | CTCAE 3.0 | Cycle |  | 20/145 | 12/145 | 8/145 |  | 29/145 | 22/145 | 7/145 |  | 0/35 |
|  |  |  |  |  |  |  |  |  |  |  |  |  |
|  |  |  |  |  |  |  |  |  |  |  |  |  |
| **Somatostatin analogs** |  |  |  |  |  |  |  |  |  |  |  |  |
| Chamberlain (2007)^17^ | NIH | Patient |  | 0/16 | 0/16 | 0/16 |  | 3/16 | 3/16 | 0/16 |  | 0/11 |
|  |  |  |  |  |  |  |  |  |  |  |  |  |
|  |  |  |  |  |  |  |  |  |  |  |  |  |
| Johnson (2011)^18^ | NCI 1.0 | Patient |  | 0/12 | 0/12 | 0/12 |  | 9/12 | 9/12 | 0/12 |  | 0/12 |
|  |  |  |  |  |  |  |  |  |  |  |  |  |
|  |  |  |  |  |  |  |  |  |  |  |  |  |
| Schulz (2011)^19^ | NA | NR |  | – | – | – |  | – | – | – |  | 1/8 |
|  |  |  |  |  |  |  |  |  |  |  |  |  |
|  |  |  |  |  |  |  |  |  |  |  |  |  |
| Simo (2014)^20^ | CTCAE 3.0 | Patient |  | 0/9 | 0/9 | 0/9 |  | 5/9 | 5/9 | 0/9 |  | 0/9 |
|  |  |  |  |  |  |  |  |  |  |  |  |  |
|  |  |  |  |  |  |  |  |  |  |  |  |  |
| Norden (2015)^21^ | CTCAE 3.0 | Patient/ cycle |  | 4/34 | 4/34 | 0/34 |  | 112/NR | 112/NR | 112/NR |  | 2/34 |
|  |  |  |  |  |  |  |  |  |  |  |  |  |
|  |  |  |  |  |  |  |  |  |  |  |  |  |
| **Temozolomide** |  |  |  |  |  |  |  |  |  |  |  |  |
| Chamberlain (2004)^22^ | CTCAE 3.0 | Patient |  | 3/16 | – | 3/16 |  | 13/16 | – | 13/16 |  | 0/16 |
|  |  |  |  |  |  |  |  |  |  |  |  |  |
|  |  |  |  |  |  |  |  |  |  |  |  |  |
| Belanger (2022)^23^ | NR | Patient |  | 2/11 | – | – |  | 7/11 | – | – |  | 0/11 |
|  |  |  |  |  |  |  |  |  |  |  |  |  |

**Supplementary Table S1 contd.** Reported hematologic and non-hematologic toxicities, stratified by grade and whether measured per patient or per treatment cycle, across included studies

|  |  |  |  |  |  |  |  |  | | |  |  |
| --- | --- | --- | --- | --- | --- | --- | --- | --- | --- | --- | --- | --- |
| **Study (Year)** | **Toxic criteria** | **Level reported** |  | **Hematologic toxicity** | | |  | **Non-hematologic toxicity** | | |  | **Discontinued treatment due to toxicity** |
|  |  |  |  |  |  |  |  |  | | |  |  |
|  |  |  |  | **Any toxicity** | **Grades I–II** | **Grades ≥3** |  | **Any toxicity** | **Grades I–II** | **Grades ≥3** |  |  |
|  |  |  |  |  |  |  |  |  |  |  |  |  |
|  |  |  |  |  |  |  |  |  |  |  |  |  |
| **PD-L1 inhibitors** |  |  |  |  |  |  |  |  |  |  |  |  |
| Bi (2022)^24^ | CTCAE 4.0 | Patient/ cycle |  | 0/25 | 0/25 | 0/25 |  | 32/NR | 22/NR | 10/NR |  | 1/25 |
|  |  |  |  |  |  |  |  |  |  |  |  |  |
|  |  |  |  |  |  |  |  |  |  |  |  |  |
| Brastianos (2022)^25^ | CTCAE 5.0 | Patient/ cycle |  | 7/NR | 7/NR | 0/NR |  | 123/NR | 110/NR | 13/NR |  | 4/23 |
|  |  |  |  |  |  |  |  |  |  |  |  |  |
|  |  |  |  |  |  |  |  |  |  |  |  |  |
| **TKIs** |  |  |  |  |  |  |  |  |  |  |  |  |
| Horak (2012)^26^ | NR | **–** |  | – | – | – |  | – | – | – |  | – |
|  |  |  |  |  |  |  |  |  |  |  |  |  |
|  |  |  |  |  |  |  |  |  |  |  |  |  |
| Raizer (2014)^27^ | NR | Patient/ cycle |  | 3/25 | 2/25 | 1/25 |  | 57/127 | 39/127 | 18/127 |  | 5/25 |

**Supplementary Table S2. Toxicity grading harmonization across systems**

|  |  |  |  |  |
| --- | --- | --- | --- | --- |
| **Toxicity** | **WHO Grade 3** | **CTCAE v2.0–4.0 Grade 3** | **CTCAE v5.0** | **Harmonized definition** |
|  |  |  |  |  |
|  |  |  |  |  |
| **Hematologic toxicity** |  |  |  |  |
| **Neutropenia** | **ANC <1.0 ×10⁹/L** | **ANC <1.0–0.5 ×10⁹/L** | **ANC <1.0–0.5 ×10⁹/L** | **Grade ≥3** |
|  |  |  |  |  |
|  |  |  |  |  |
| **Leukopenia** | **WBC <2.0 ×10⁹/L** | **WBC <2.0–1.0 ×10⁹/L** | **WBC <2.0–1.0 ×10⁹/L** | **Grade ≥3** |
|  |  |  |  |  |
|  |  |  |  |  |
| **Thrombocytopenia** | **<50 ×10⁹/L** | **<50 ×10⁹/L** | **<50 ×10⁹/L** | **Grade ≥3** |
|  |  |  |  |  |
|  |  |  |  |  |
| **Anemia** | **Hb <8.0 g/dL** | **Hb <8.0 g/dL** | **Hb <8.0 g/dL** | **Grade ≥3** |
|  |  |  |  |  |
|  |  |  |  |  |
| **Pancytopenia** | **Severe multilineage suppression** | **Severe multilineage suppression** | **Severe multilineage suppression** | **Grade ≥3** |
|  |  |  |  |  |
|  |  |  |  |  |
| **Metabolic and laboratory abnormalities** | |  |  |  |
| **Hyperglycemia** | **>250 mg/dL** | **>250 mg/dL** | **Insulin therapy initiated;**  **hospitalization indicated** | **Grade ≥3** |
|  |  |  |  |  |
|  |  |  |  |  |
| **Hypertriglyceridemia** | **Severe elevation** | **Severe elevation; hosptilization needed** | **>500–1000 mg/dL** | **Grade ≥3** |
|  |  |  |  |  |
|  |  |  |  |  |
| **LFT elevation** | **>5× ULN** | **>5–20× ULN** | **>5–20× ULN** | **Grade ≥3** |
|  |  |  |  |  |
|  |  |  |  |  |
| **Hyperuricemia** | **Severe elevation** | **Severe elevation** | **>ULN with physiologic**  **consequences** | **Grade ≥3** |
|  |  |  |  |  |

**For symptom-based toxicities (e.g., fatigue, nausea, diarrhea, rash), grade ≥3 was defined consistently across systems as severe symptoms limiting self-care activities of daily living, requiring medical intervention, or hospitalization. Life-threatening or medically significant events (e.g., pulmonary embolism, GI perforation, major bleeding) were uniformly classified as grade ≥3 across systems.**

**Supplementary Table S3.** Pooled estimates of hematologic and non-hematologic toxicities, summarized by drug class, presented as proportions with 95% confidence intervals.

|  |  |  |  |  |
| --- | --- | --- | --- | --- |
| **Toxicity** | **Drug Class** | | | |
|  |  |  |  |  |
|  | **Bevacizumab** | **Hydroxyurea** | **INF-α** | **Somatostatin analogs** |
|  |  |  |  |  |
|  |  |  |  |  |
| **Hematologic toxicity** |  |  |  |  |
| *Events per patient* |  |  |  |  |
| Any toxicity | 0.50% (0.0–48.0) | 67.36% (26.3–92.3) | – | 2.6% (0.08–47.4) |
| Grade I/II toxicity | 0.99 (0.03–24.82) | 38.7% (20.8–60.4) | – | 2.6% (0.08–47.4) |
| Grade ≥3 toxicity | 0.99 (0.03–24.82) | 11.7% (2.0–46.0) | – | – |
|  |  |  |  |  |
|  |  |  |  |  |
| *Events per cycle* |  |  |  |  |
| Any toxicity | – | 13.5% (11.1–16.2,) | 11.9% (9.0–15.5) | – |
| Grade I/II toxicity | – | 12.2% (10.0–14.8) | 8.0% (5.7–11.2) | – |
| Grade ≥3 toxicity | – | 1.3% (0.7–2.4) | 3.6% (2.2–6.0) | – |
|  |  |  |  |  |
|  |  |  |  |  |
| **Non-hematologic toxicity** |  |  |  |  |
| *Events per patient* |  |  |  |  |
| Any toxicity | – | 3.9% (1.0–14.12) | – | 48.3% (20.6–77.0) |
| Grade I/II toxicity | – | 3.9% (1.0–14.12) | – | 48.3% (20.6–77.0) |
| Grade ≥3 toxicity | – | – | – | – |
|  |  |  |  |  |
|  |  |  |  |  |
| *Events per cycle* |  |  |  |  |
| Any toxicity | – | 17.1% (8.9–30.4) | 17.6% (14.1–21.7) | – |
| Grade I/II toxicity | – | 15.5% (8.1–27.8) | 14.2% (11.1–18.1) | – |
| Grade ≥3 toxicity | – | 1.1 (0.6–2.3) | 3.6% (2.2–6.0) | – |
|  |  |  |  |  |
|  |  |  |  |  |
| Discontinuation due to toxicity | 16.2% (10.5–24.3) | 3.0% (0.9–9.8) | 3.3% (0.4–24.0) | 4.1% (1.3–11.8) |
|  |  |  |  |  |

**Supplementary Table S4.** Pharmacological mechanisms of action of drugs used in study.

|  |  |
| --- | --- |
| **Drug agent/class** | **Mechanism of action** |
|  |  |
|  |  |
| Hydroxyurea | The cytotoxic antimetabolite hydroxyurea inhibits ribonucleotide reductase and DNA synthesis, producing occasional transient stabilization but inconsistent benefit and notable hematologic toxicity.^13,28^ |
|  |  |
|  |  |
| Somatostain analogs | Somatostatin analogs act on somatostatin receptor 2A (SSTR2A)-positive tumour cells to suppress cyclic-AMP signalling and growth-factor release, with prior clinical studies yielding variable, largely non-durable responses. |
|  |  |
|  |  |
| INF-α | IFN-α exerts antiproliferative and anti-angiogenic activity through JAK/STAT pathway activation, up-regulation of the cell-cycle inhibitor p21, and suppression of VEGF/FGF-mediated endothelial signaling, with additional immune-modulatory effects that together yield a cytostatic phenotype rather than tumor regression.^29,30^ |
|  |  |
|  |  |
| Bevacizumab | Anti-vascular endothelial growth factor (VEGF) antibodies such as bevacizumab inhibit angiogenesis by neutralizing VEGF-A and reducing tumor vascular permeability.^3^ Mechanistically, bevacizumab neutralizes VEGF-A and attenuates VEGFR-1/2 signaling, leading to vascular normalization, reduced permeability, and decreased peritumoral edema; effects that explain the predominance of radiographic stability and symptomatic relief over frank cytoreduction.^31,32^ |
|  |  |
|  |  |
| Everolimus | mTOR inhibitors such as everolimus act downstream, forming an FKBP12-everolimus complex that allosterically inhibits mTORC1 and reduces protein synthesis and VEGF expression.^33^ |
|  |  |
|  |  |
| Tyrosine kinase inhibitors | Broad receptor-tyrosine-kinase inhibition (VEGFR/PDGFR/KIT) attenuates angiogenesis and downstream PI3K-AKT-mTOR and RAS-RAF-MEK-ERK signaling, producing a cytostatic, stabilization-dominant phenotype.^34^ Multi-targeted tyrosine-kinase inhibitors (TKIs) including sunitinib block VEGFR, PDGFR, and KIT phosphorylation, thereby suppressing PI3K/AKT/mTOR and MAPK signaling.^34^ |
|  |  |
|  |  |
| PD-1/PD-L1 blockers | By preventing PD-1 interaction with PD-L1, agents such as pembrolizumab and nivolumab reinvigorate exhausted cytotoxic T-cells and enhance immune-mediated tumor control.^35,36^ High-grade meningiomas frequently express PD-L1 and exhibit immune-suppressive microenvironments. [65] PD-1/PD-L1 blockade re-engages exhausted cytotoxic T cells within PD-L1-expressing meningiomas, whose immune-suppressive microenvironments intensify with grade.^35,37^ |
|  |  |

**References**

1. Automeris. WebPlotDigitizer. *https://automeris.io/*.

2. Guyot, P., Ades, A. E., Ouwens, M. J. N. M. & Welton, N. J. Enhanced secondary analysis of survival data: reconstructing the data from published Kaplan-Meier survival curves. *BMC Med. Res. Methodol.* **12**, 9 (2012).

3. Lou, E. *et al.* Bevacizumab therapy for adults with recurrent/progressive meningioma: a retrospective series. *J. Neurooncol.* **109**, 63–70 (2012).

4. Nayak, L. *et al.* Atypical and anaplastic meningiomas treated with bevacizumab. *J. Neurooncol.* **109**, 187–93 (2012).

5. Shih, K. C. *et al.* A phase II trial of bevacizumab and everolimus as treatment for patients with refractory, progressive intracranial meningioma. *J. Neurooncol.* **129**, 281–8 (2016).

6. Alexander, A. Y. *et al.* The role of bevacizumab for treatment-refractory intracranial meningiomas: a single institution’s experience and a systematic review of the literature. *Acta Neurochir. (Wien).* **164**, 3011–3023 (2022).

7. Kumthekar, P. *et al.* A multi-institutional phase II trial of bevacizumab for recurrent and refractory meningioma. *Neurooncol. Adv.* **4**, vdac123 (2022).

8. Mason, W. P. *et al.* Stabilization of disease progression by hydroxyurea in patients with recurrent or unresectable meningioma. *J. Neurosurg.* **97**, 341–6 (2002).

9. Loven, D. *et al.* Non-resectable slow-growing meningiomas treated by hydroxyurea. *J. Neurooncol.* **67**, 221–6 (2004).

10. Newton, H. B., Scott, S. R. & Volpi, C. Hydroxyurea chemotherapy for meningiomas: enlarged cohort with extended follow-up. *Br. J. Neurosurg.* **18**, 495–9 (2004).

11. Hahn, B. M. *et al.* Prolonged oral hydroxyurea and concurrent 3d-conformal radiation in patients with progressive or recurrent meningioma: results of a pilot study. *J. Neurooncol.* **74**, 157–65 (2005).

12. Chamberlain, M. C. Hydroxyurea for recurrent surgery and radiation refractory high-grade meningioma. *J. Neurooncol.* **107**, 315–21 (2012).

13. Kim, M.-S. *et al.* Long-term follow-up result of hydroxyurea chemotherapy for recurrent meningiomas. *J. Korean Neurosurg. Soc.* **52**, 517–22 (2012).

14. Karsy, M. *et al.* Combined Hydroxyurea and Verapamil in the Clinical Treatment of Refractory Meningioma: Human and Orthotopic Xenograft Studies. *World Neurosurg.* **86**, 210–9 (2016).

15. Kim, J., Kim, K. H. & Kim, Y. Z. The Clinical Outcome of Hydroxyurea Chemotherapy after Incomplete Resection of Atypical Meningiomas. *Brain Tumor Res. Treat.* **5**, 77–86 (2017).

16. Chamberlain, M. C. & Glantz, M. J. Interferon-alpha for recurrent World Health Organization grade 1 intracranial meningiomas. *Cancer* **113**, 2146–51 (2008).

17. Chamberlain, M. C., Glantz, M. J. & Fadul, C. E. Recurrent meningioma: salvage therapy with long-acting somatostatin analogue. *Neurology* **69**, 969–73 (2007).

18. Johnson, D. R. *et al.* Phase II study of subcutaneous octreotide in adults with recurrent or progressive meningioma and meningeal hemangiopericytoma. *Neuro. Oncol.* **13**, 530–5 (2011).

19. Schulz, C., Mathieu, R., Kunz, U. & Mauer, U. M. Treatment of unresectable skull base meningiomas with somatostatin analogs. *Neurosurg. Focus* **30**, E11 (2011).

20. Simó, M. *et al.* Recurrent high-grade meningioma: a phase II trial with somatostatin analogue therapy. *Cancer Chemother. Pharmacol.* **73**, 919–23 (2014).

21. Norden, A. D. *et al.* Phase II study of monthly pasireotide LAR (SOM230C) for recurrent or progressive meningioma. *Neurology* **84**, 280–6 (2015).

22. Chamberlain, M. C., Tsao-Wei, D. D. & Groshen, S. Temozolomide for treatment-resistant recurrent meningioma. *Neurology* **62**, 1210–2 (2004).

23. Belanger, K., Ung, T. H., Damek, D., Lillehei, K. O. & Ormond, D. R. Concomitant Temozolomide plus radiotherapy for high-grade and recurrent meningioma: a retrospective chart review. *BMC Cancer* **22**, 367 (2022).

24. Bi, W. L. *et al.* Activity of PD-1 blockade with nivolumab among patients with recurrent atypical/anaplastic meningioma: phase II trial results. *Neuro. Oncol.* **24**, 101–113 (2022).

25. Brastianos, P. K. *et al.* Phase 2 study of pembrolizumab in patients with recurrent and residual high-grade meningiomas. *Nat. Commun.* **13**, 1325 (2022).

26. Horak, P. *et al.* Imatinib mesylate treatment of recurrent meningiomas in preselected patients: a retrospective analysis. *J. Neurooncol.* **109**, 323–30 (2012).

27. Raizer, J. J. *et al.* A phase II trial of PTK787/ZK 222584 in recurrent or progressive radiation and surgery refractory meningiomas. *J. Neurooncol.* **117**, 93–101 (2014).

28. Schrell, U. M. *et al.* Hydroxyurea for treatment of unresectable and recurrent meningiomas. II. Decrease in the size of meningiomas in patients treated with hydroxyurea. *J. Neurosurg.* **86**, 840–4 (1997).

29. Borden, E. C. *et al.* Interferons at age 50: past, current and future impact on biomedicine. *Nat. Rev. Drug Discov.* **6**, 975–90 (2007).

30. Chamberlain, M. C. IFN-α for recurrent surgery- and radiation-refractory high-grade meningioma: a retrospective case series. *CNS Oncol.* **2**, 227–35 (2013).

31. Ferrara, N., Hillan, K. J., Gerber, H.-P. & Novotny, W. Discovery and development of bevacizumab, an anti-VEGF antibody for treating cancer. *Nat. Rev. Drug Discov.* **3**, 391–400 (2004).

32. Jain, R. K. *et al.* Angiogenesis in brain tumours. *Nat. Rev. Neurosci.* **8**, 610–622 (2007).

33. Zoncu, R., Efeyan, A. & Sabatini, D. M. mTOR: from growth signal integration to cancer, diabetes and ageing. *Nat. Rev. Mol. Cell Biol.* **12**, 21–35 (2011).

34. Faivre, S., Demetri, G., Sargent, W. & Raymond, E. Molecular basis for sunitinib efficacy and future clinical development. *Nat. Rev. Drug Discov.* **6**, 734–45 (2007).

35. Topalian, S. L. *et al.* Safety, Activity, and Immune Correlates of Anti–PD-1 Antibody in Cancer. *New England Journal of Medicine* **366**, 2443–2454 (2012).

36. Pardoll, D. M. The blockade of immune checkpoints in cancer immunotherapy. *Nat. Rev. Cancer* **12**, 252–264 (2012).

37. Han, S. J. *et al.* Expression and prognostic impact of immune modulatory molecule PD-L1 in meningioma. *J. Neurooncol.* **130**, 543–552 (2016).
